# An Off‐the‐Shelf Artificial Proregenerative Macrophage for Pressure Ulcer Treatment

**DOI:** 10.1002/advs.202415886

**Published:** 2025-04-24

**Authors:** Qi Su, Jingrong Wang, Yini Huangfu, Rui Gao, Pengxu Kong, Yu Gao, Huijuan Song, Ju Zhang, Pingsheng Huang, Chuangnian Zhang, Zujian Feng, Deling Kong, Weiwei Wang

**Affiliations:** ^1^ State Key Laboratory of Advanced Medical Materials and Devices Institute of Biomedical Engineering Chinese Academy of Medical Sciences and Peking Union Medical College Tianjin 300192 China; ^2^ Beijing Life Science Academy Beijing 102200 China; ^3^ Department of Thoracic Surgery The First Affiliated Hospital School of Medicine Zhejiang University Hangzhou 310006 China; ^4^ Institute of Radiation Medicine Chinese Academy of Medical Sciences and Peking Union Medical College Tianjin 300192 China; ^5^ College of Life Sciences Key Laboratory of Bioactive Materials (Ministry of Education) State Key Laboratory of Medicinal Chemical Biology Nankai University Tianjin 300071 China

**Keywords:** bioactive materials, inflammation modulation, macrophages, pressure ulcer, tissue regeneration

## Abstract

Cell therapy is a promising approach in regenerative medicine. However, maintaining the survival and function of injected or implanted therapeutic cells remains a substantial challenge to success. In vivo modulatory strategy for cell therapeutics has been recently developed, but suffers from limited regenerative efficacy in injured tissue microenvironment with chronic inflammation. Here, an off‐the‐shelf artificial macrophage (artM) assembled by M2 macrophages‐derived lysate proteins‐loaded poly (lactic‐co‐glycolic acid) (PLGA) microspheres coated by macrophage cell membrane is developed. The synthetic artM fabricated in batches maintains its bioactivity with long‐term cryostorage. Significantly, artM recapitulates the essential inflammation‐regulatory and proregenerative characteristics of endogenous macrophages, including initiating M2 macrophage polarization, resolving excessive inflammation by releasing anti‐inflammatory cytokines and growth factors, neutralizing endotoxins and proinflammatory cytokines, augmenting T‐helper 2 (T_H_2) immune response, and coordinating cell migration and proliferation. In mouse model of deep tissue pressure injury (DTPI), the artM induces tissue regeneration by modulating the inflammatory microenvironment, promoting angiogenesis, reducing scar deposition, and accelerating the renewal of skin appendages. Depletion of macrophages in mice with skin ulcers highlights the immunomodulatory and proangiogenic functions of artM as effective as autogenous macrophages. Collectively, the engineered artM represents a cell‐free, proreparative alternative to immune cell therapy in chronic wound management.

## Graphical abstract

Artificial macrophages (artM) by encapsulating M2 lysates into biodegradable PLGA microspheres and coated with macrophage membrane, are engineered as new therapeutics against skin pressure ulcer. In mouse models, the artM exhibited robust tissue repair through cell recruitment and microenvironment modulation, showing great therapeutic potential for chronic wound healing and tissue regeneration as an off‐the‐shelf alternative of cell therapy.

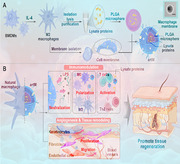

## 1 Introduction

Regenerative medicine uses live therapeutic cells to repair or replace damaged organs or tissues via injection or implantation into a patient.^[^ ^1^ ^]^ Bone marrow‐ or adipose‐derived stem cells, and immune cells including T cells and macrophages, have been adopted for in vivo transplantation after modification in vitro.^[^ ^2^ ^]^ Specially, macrophages, a subset of phagocytes within the innate immune system, are derived from monocytes and greatly involved in maintaining tissue homeostasis and defending against diseases.^[^ ^3^ ^]^ It is being increasingly recognized that anti‐inflammatory M2 macrophages are closely related to cell proliferation and differentiation, angiogenesis, and tissue remodeling during wound healing, and their dysfunction may cause chronic inflammation and difficult‐to‐healing wounds.^[^ ^3^, ^4^ ^]^ A typical case is deep tissue pressure injury (DTPI), a serious pressure ulcer that imposes a heavy medical and economic burden annually.^[^ ^5^ ^]^ For DTPI, proinflammatory M1 macrophages fail to transit into anti‐inflammatory states, while secrete proinflammatory chemo‐ and cytokines, such as monocyte chemoattractant protein‐1 (MCP‐1) and interleukin‐6 (IL‐6), to further deteriorate the inflammation in the wound site.^[^ ^6^ ^]^ Ex vivo‐modified regenerative M2 macrophages have thus been designed in preclinical studies and clinical trials to improve the healing of chronic wounds, which outperformed stem cells in some cases.^[^ ^7^ ^]^ Moreover, the recently developed chimeric antigen receptor‐macrophages (CAR‐M) revealed the potential of genetically engineered macrophages for cell therapy.^[^ ^8^ ^]^ However, adoptive transfer of M2 macrophages confronts with challenges commonly present in cell therapy, including sophisticated modification procedure, long‐term storage, massive loss of cell viability and function, and low engraftment efficiency after cell transplantation.^[^ ^9^ ^]^ Furthermore, macrophages are highly plastic and heterogeneous, which can rapidly change their functions in response to local microenvironmental cues; thus, it is quite tough for macrophages to maintain their regenerative functions after transfusion in vivo, especially in the chronic inflammation microenvironment of injured tissue.^[^ ^10^ ^]^

Alternatively, great efforts have been focused on promoting polarization of endogenous macrophages or monocytes into M2 phenotype for tissue regeneration in recent years. Delivering cytokines such as recombinant IL‐4 or IL‐10, or anti‐inflammatory drugs has been used to recruit endogenous macrophages and induce proregenerative function or block proinflammatory signaling pathways, respectively.^[^ ^11^ ^]^ However, the efficacy of these modulatory approaches for cell therapeutics is limited by uncontrollable delivery efficiency, rapid diffusion or degradation, and adverse effects associated with cytokines or drugs. Moreover, the aberrant inflammatory microenvironment with excessive amounts of proteases and reactive oxygen species also compromised the therapeutic effectiveness of polarized macrophages. More recently, immunomodulatory biomaterials emerged to promote tissue regeneration on their own by regulating the immune response in inflammatory situations with tissue damage.^[^ ^12^ ^]^ By mimicking the extracellular matrix, biomaterial scaffolds can serve as an immunomodulatory niche to reconstruct a prohealing immune environment by coordinating macrophage/T‐cell responses and stromal cell migration and proliferation, therefore achieving endogenous tissue repair.^[^ ^13^ ^]^ However, the material component (e.g., natural versus synthetic), scaffold physical properties (e.g., surface roughness, microstructure and stiffness), and additional biochemical cues (e.g., cellular ligands) may profoundly impact the immune cell differentiation, which have not yet been fully elucidated.^[^ ^14^ ^]^ Thus, although macrophage regulation by *ex vivo* genetically engineered technology, or in vivo stimulating approach using biomaterials with or without immunomodulators is promising, it remains a great challenge to accurately manipulate the macrophage phenotypes and functions to develop cell‐free, highly efficient therapeutics for tissue regeneration.

**Figure 1:**
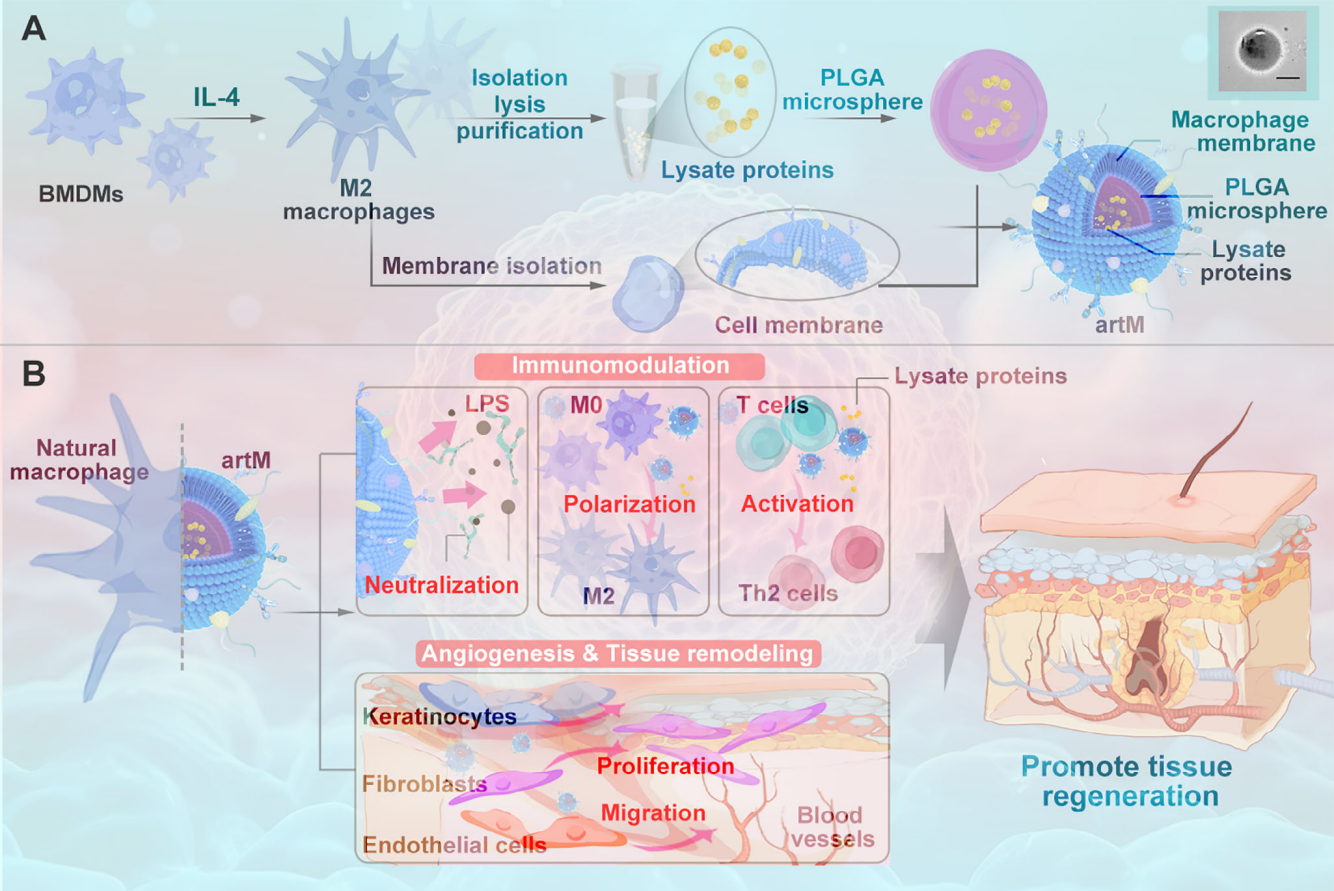
Schematic illustration of the fabrication and biofunctions of engineered artM in promoting skin regeneration of DTPI. A) The artM was assembled by M2 macrophage‐derived lysate protein‐loaded PLGA microspheres coated with macrophage membrane. B) The off‐the‐shelf artM was feasible for cryopreservation and showed the essential functions of endogenous prohealing macrophages, including immunomodulation via neutralizing proinflammatory cytokines, polarizing M2 macrophages and activating T_H_2 response, and proregeneration mediated by angiogenesis and tissue remodeling. Altogether, the artM provided an alternative cell‐free therapeutic for DTPI management.

Precisely engineered materials were being increasingly developed to fabricate artificial cells that can replicate the cellular morphology and biological function, as an alternative to living cells. Here, to bypass the high plasticity and heterogeneity of live macrophages, we synthesized an artificial macrophage (artM) with bottom‐up design strategy, which was expected to recapitulate and maintain the cellular function of living macrophages and to shape the regeneration of impaired tissue. As shown in **Figure** **1**, bioactive proteins were first isolated, purified and collected from the lysate of IL‐4‐induced M2 macrophages. Then, the artM was fabricated by encapsulating lysate proteins into biodegradable poly (lactic‐co‐glycolic acid) (PLGA) microspheres, and further coated with cell membrane from macrophages. The embedded proteins comprise anti‐inflammatory cytokines and prohealing growth factors, which may be sustainedly released from the microspheres and impart artM proregenerative bioactivities in vivo, including improving cell recruitment and proliferation, alleviating inflammation, and promoting angiogenesis and tissue reconstruction. The integration of polymer microspheres and cell membrane not only mimics the cell morphology and lipid layer of native macrophages, but also contribute to reserve cellular functions. Moreover, all components in artM are acellular, potentially overcoming the shortages of regenerative therapy using alive cells and providing an off‐the‐shelf product with autogenous macrophage alike biofunctions, which may broaden the application of cell‐based therapy in clinic. We tested the physicochemical and biological properties of the artM in vitro and detected its treatment benefits in mouse models of DTPI. By revealing cell biology, the artM provides an acellular, off‐the‐shelf, and in vivo proreparative therapeutics for chronic wound healing, even in the absence of endogenous macrophages. The artM may be also applicable for the repair of other tissue injures, such as cardiac damage, muscle defect and diabetic ulcer.

## 2 Results

### 2.1 Preparation and Characterization of artM

**Figure 2:**
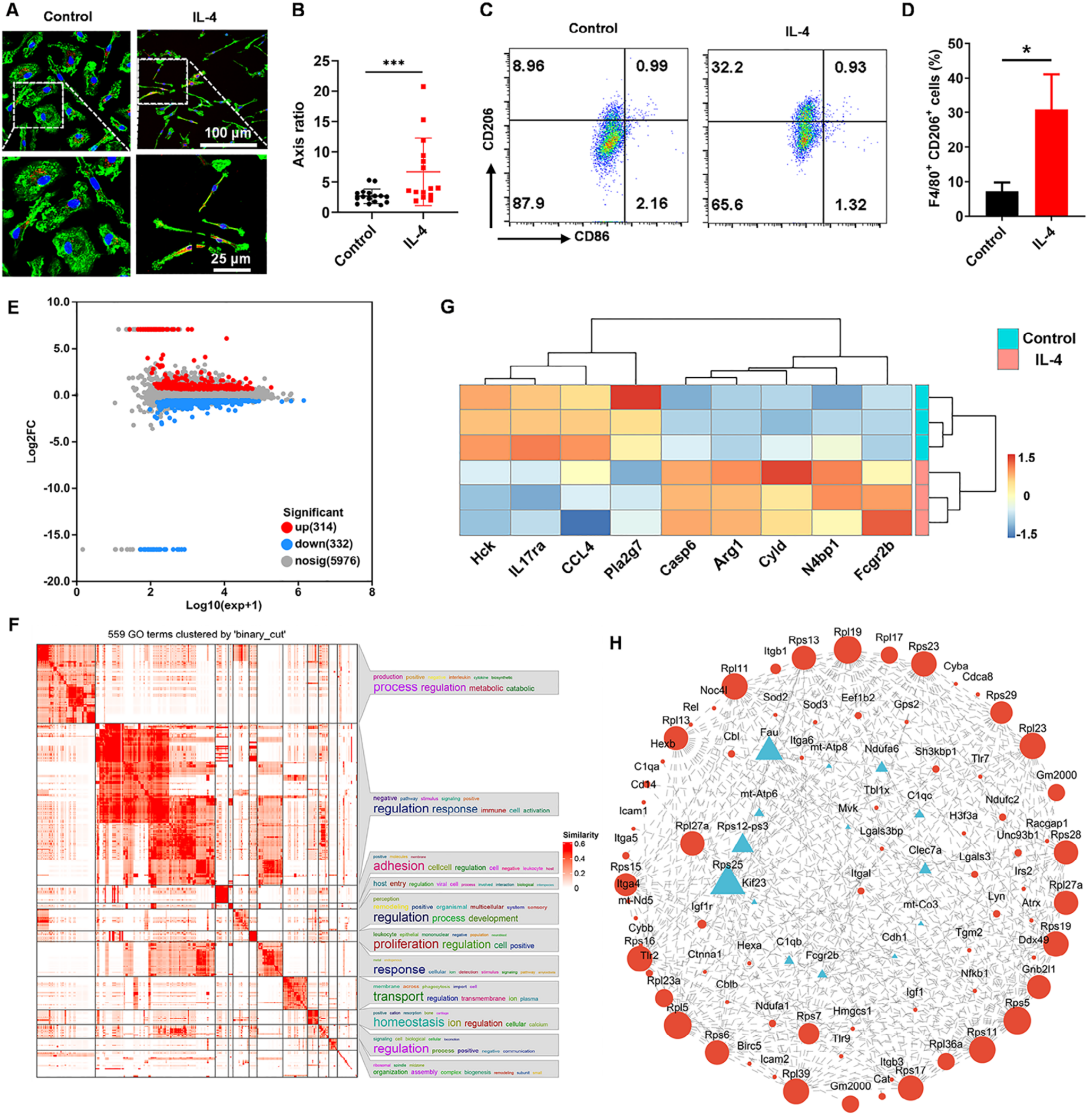
Preparation and identification of lysate from M2 macrophages. A) Representative CLSM images of BMDMs treated with PBS or IL‐4 for 48 h (red: CD206; green: F‐actin; blue: cell nuclear). B) The axis ratio of treated BMDMs. C) Flow cytometry analysis of CD206 and CD86 expression of BMDMs (gated on F4/80^+^ cells). D) Percentage of M2 (F4/80^+^CD206^+^) type macrophages (*n* = 3). E) Volcano map of protein expression in M2 macrophages compared with primary BMDMs. Red and blue points represent upregulated and downregulated proteins selected based on *p*‐value < 0.05 and |logFC| > 2, respectively, while gray points represent proteins with no significant difference. F) GO functional enrichment on the DEPs, with p‐value < 0.05 indicating significance. The terms of significant biological processes (BP) were clustered, and the functions of clustered terms were shown in the right column. G) The cluster heat map of DEPs in the top GO term. H) PPI network of DEPs.

To prepare anti‐inflammatory M2 macrophages, primary bone marrow‐derived macrophages (BMDMs) were isolated from bone marrow monocytes through incubation with macrophage colony‐stimulating factor (M‐CSF), and further stimulation by IL‐4, a typical anti‐inflammatory cytokine driving M2 macrophages activation through the JAK/STAT6 signaling pathway. First, changes in cell morphology were identified by confocal laser scanning microscopy (CLSM) through labeling F‐actin with FITC‐conjugated phalloidine and characteristic biomarker CD206 of M2 macrophages. As shown in **Figure** **2**A, polarized BMDMs induced by IL‐4 showed elongated and pseudopod‐like structures, the typical cell morphology of M2 phenotype, and stronger red fluorescence of CD206 staining, compared to untreated BMDMs. The axis ratio of cells also demonstrated that IL‐4 treatment resulted in elongated structures (Figure 2B). Flow cytometry (Figure 2C,D) demonstrated that after gated with F4/80^+^ cells, the percentage of M2 type (F4/80^+^CD206^+^) macrophages was significantly elevated after IL‐4 treatment, indicating the successful polarization of macrophages into M2 phenotype. Subsequently, proteins of M2 macrophage lysate were obtained through freeze‐thawing in liquid nitrogen for 3 times, and the supernatant was collected by centrifugation for further purification by protein extraction kit according to the standard protocols. To identify the type and function of proteins in M2 macrophage lysate, IL‐4 treated or untreated samples were subjected to proteomics analysis. As shown in Figure 2E, a total of 646 differently expressed proteins (DEPs) were selected according to the criteria (*p*‐value < 0.05 and |logFC| > 2), including 314 and 332 proteins were upregulated and downregulated, respectively. Then, DEPs were functionally annotated and enriched. Figure 2F showed that the functions of these DEPs could be significantly enriched in terms of immune cell activation, metabolic regulation, cell proliferation and membrane transport, with a higher enrichment in immune cell activation. The DEPs in the top Gene Ontology (GO) term were visualized by cluster heat map, and the expression of hematopoietic cell‐dominated nonreceptor tyrosine protein kinase (Hck), interleukin‐17 receptor a (IL17Ra), C‐C motif chemokine 4 (CCL4) and platelet‐activating factor acetylhydrolase (PLa2g7) related to proinflammation were decreased, while the expression of DEPs related to anti‐inflammation such as arginase‐1 (Arg‐1) were increased (Figure 2G). In addition, the protein–protein interaction (PPI) network of DEPs was constructed through the STRING database, which was composed of 88 nodes and 300 edges (Figure 2H). The PPI network was mainly divided into two modules, and the related functions of which involved inflammation and immunity. These results indicated that the biofunction of cell lysate proteins from IL‐4 treated macrophages was mainly related to immune activation, including the activation of inflammation‐related proteins and pathways, especially the inhibition of proinflammatory proteins and pathways, and the significant up‐regulation of anti‐inflammatory proteins and pathways, suggesting the presence of abundant anti‐inflammatory components in the lysate proteins.

**Figure 3:**
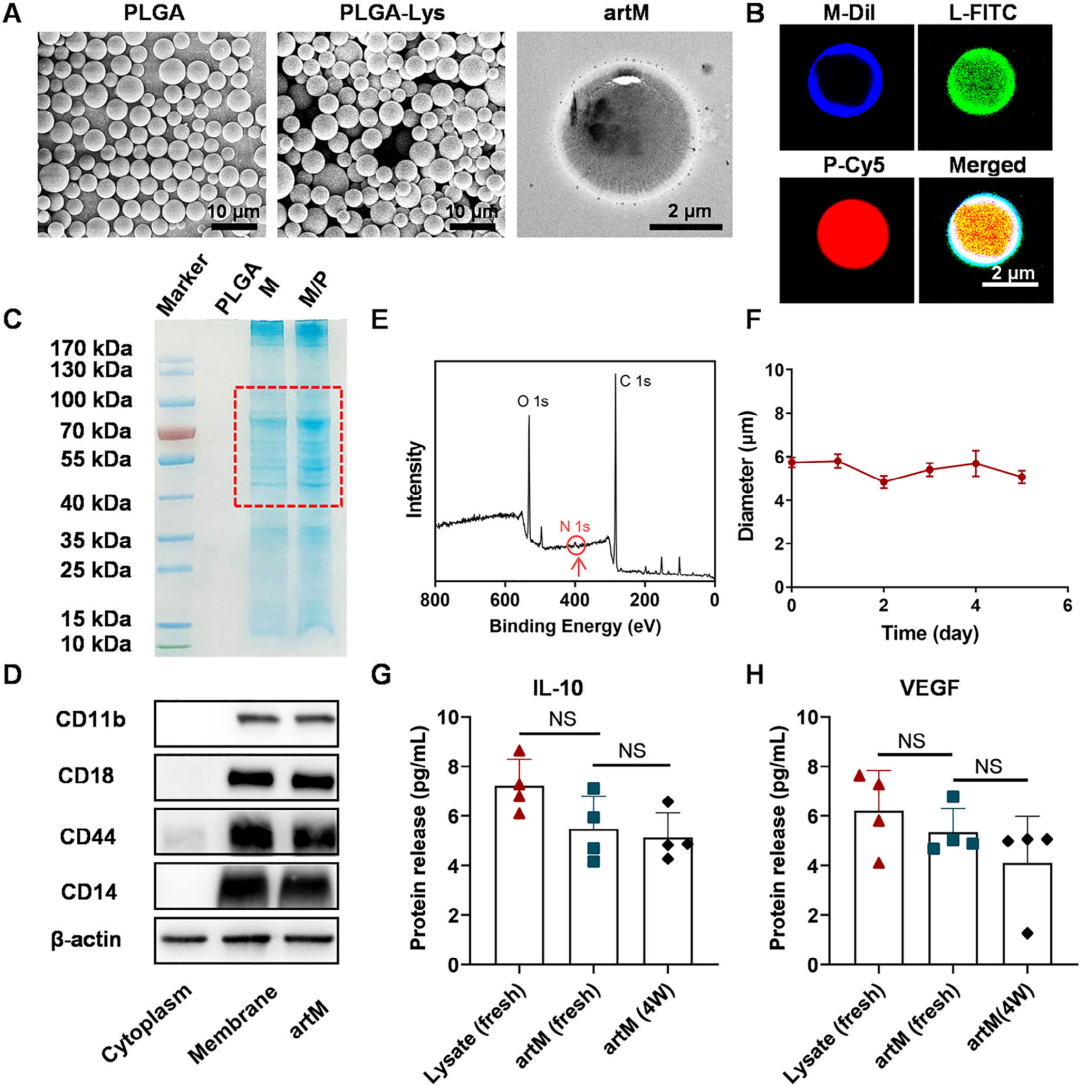
Preparation and characterization of engineered artM. A) Representative SEM images of PLGA and PLGA‐Lys, and TEM image of artM. B) Representative CLSM images of artM (blue: DiI; green: FITC; red: Cy5; M: cell membrane; L: lysate; P: PLGA). C) SDS‐PAGE of crucial membrane proteins (M: cell membrane; P: PLGA). D) Protein expression of CD11b, CD18, CD44 and CD14 determined by WB. E) The XPS spectrum of artM. F) The stability of artM in PBS (10% FBS) under storage for 5 days. G, H) IL‐10 (G) and vascular endothelial growth factor (VEGF) (H) analyzed by ELISA (*n* = 4). W: week.

To fabricate artM, the lysate of M2 macrophages were embedded in PLGA microspheres serving as the cell cytoskeleton, which was then coated by the cell membrane of M2 macrophages. Firstly, PLGA with a molecular weight of 30 kDa were selected to fabricate microspheres (terms as PLGA) and the cell lysate were encapsulated in PLGA microspheres (termed as PLGA‐Lys) through double emulsification method. As shown in **Figure** **3**A, scanning electron microscopy (SEM) indicated both PLGA and PLGA‐Lys displayed uniform spherical structure with an average diameter of ≈5 µm. The encapsulation efficiency of PLGA microspheres for the lysate was as high as 99%, as characterized by the change of protein content determined by BCA assay (Figure S1, Supporting Information) before and after encapsulation. Then, cell membrane of macrophages was isolated and coated onto PLGA‐Lys by sonication method. Representative TEM image demonstrated that the engineered artM has a spherical core‐shell structure, where PLGA core was wrapped with a thin cell membrane shell. Confocal imaging was used to further demonstrate the composition and structure of artM after staining with different dyes against each component. As shown in Figure 3B, the outer layer of artM was successfully coated with cell membrane labeled with DiI, and the inner core was Cy5‐labeled PLGA microspheres, which were loaded with cell lysate labeled by FITC. The diameter of artM was further measured by dynamic light scattering (DLS). Compared with native PLGA microspheres, the size of artM increased from 5.3 ± 0.3 µm to 5.9 ± 0.2 µm (Figure S2A, Supporting Information) due to the fusion of cell membranes. Meanwhile, artM showed a negative surface zeta potential around −1.75 mV (Figure S2B, Supporting Information), due to the presence of surface cell membranes. Moreover, the crucial membrane proteins were evaluated by sodium dodecyl sulfate polyacrylamide gel electrophoresis (SDS‐PAGE) analysis. Figure 3C demonstrated characteristic proteins, such as CD14 (52–55 kDa), CD44 (81 kDa), Mac‐1 (CD18, 85 kDa), and CD11b (128 kDa) were found in membrane/PLGA complex, further proving the successful coating of macrophage membrane. Western blot (WB) further confirmed the presence of these specific markers on the macrophage membrane (Figure 3D). Next, the chemical composition of artM surface was analyzed by X‐ray photoelectron spectroscopy (XPS). As shown in Figure 3E, the peak of N element clearly appeared, indicating the successful introduction of cell membrane on PLGA microspheres. Additionally, artM incubated in PBS containing 10% fetal bovine serum (FBS) showed good stability in size and membrane coating over 5 days (Figure 3F). These findings suggested that the artM with macrophage cell‐mimicking core‐shell structure and protein composition were successfully engineered.

One major advantage of the artM over the living macrophages is its storage stability. Specifically, cytokine secretion is an important way for macrophages to exert anti‐inflammatory function. Therefore, we evaluated the concentrations of vascular endothelial growth factor (VEGF) and anti‐inflammatory cytokine (IL‐10) released by artM after cryopreservation for 4 weeks, as they are crucial for the biological functions of macrophages. After cryopreservation, the artM was thawed, rinsed three times with sterile PBS, and soaked in 1 mL of sterile PBS for 24 h (37 °C). The concentrations of cytokines in the supernatant after centrifugation were measured using an ELISA kit. The results indicate that the factor‐releasing ability of the artM was not affected by cryopreservation (Figure 3G,H), suggesting its superiority in storage stability compared to living macrophages.

### 2.2 Anti‐Inflammatory Activities of artM

**Figure 4:**
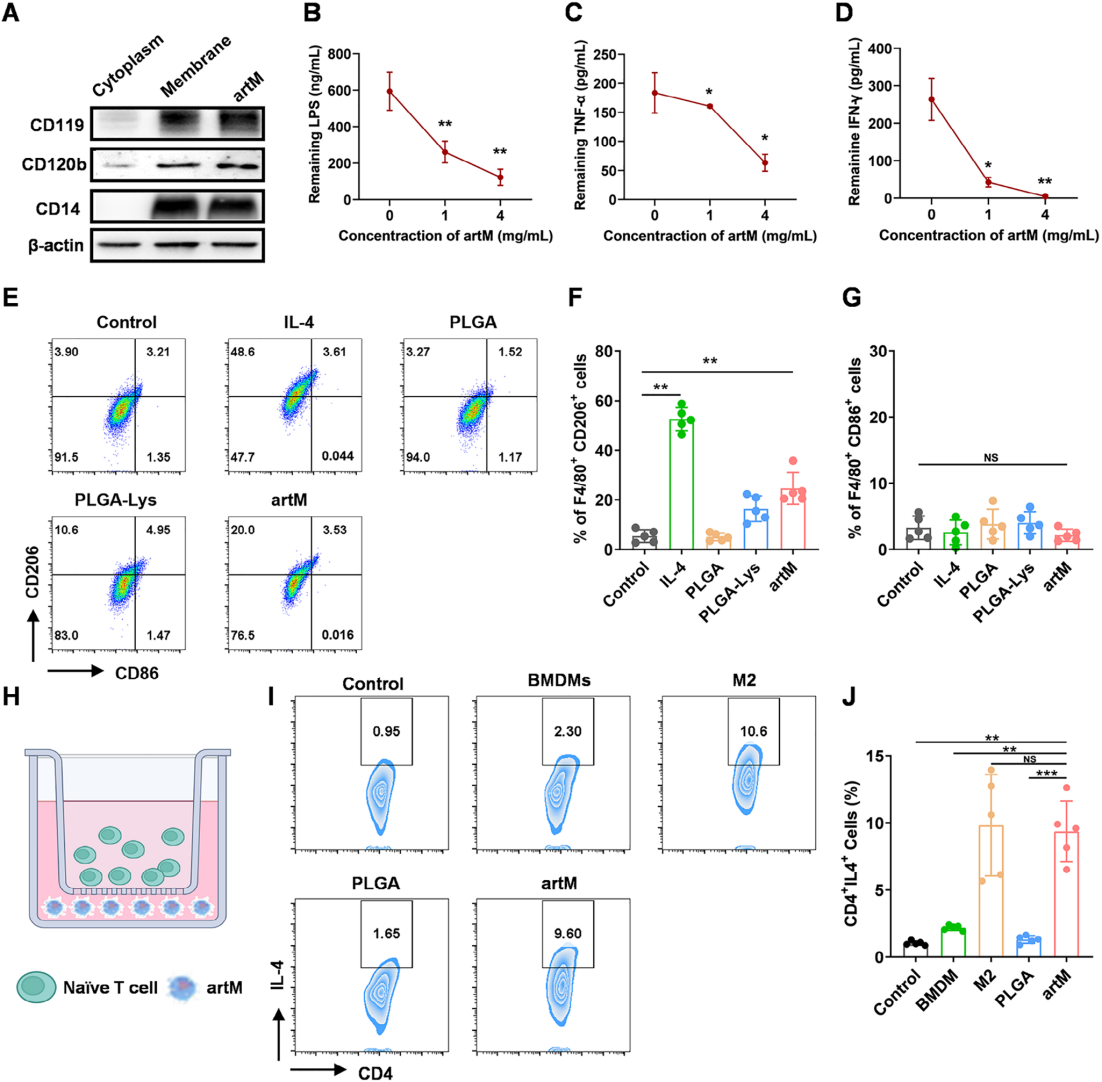
artM regulated inflammation through coordinating proinflammatory cytokine scavenging, M2 macrophages polarization and T_H_2 immune response. A) Protein expression of CD119, CD120b, and CD14 determined by WB. B–D) Remaining proinflammatory cytokines after incubated with the artM, including LPS B), TNF‐α C) and IFN‐γ D). E) Flow cytometry analysis of CD206 and CD86 expression of BMDMs after receiving different treatments (gated on F4/80^+^ cells). F,G) Percentage of F) M2 (F4/80^+^CD206^+^) and G) M1 (F4/80^+^CD86^+^) type macrophages (*n* = 5). H) Schematic illustration of the artM/lymphocytes co‐incubation system. I) Flow cytometry analysis of CD4 and IL‐4 expression of lymphocytes after receiving different treatments (gated on CD3^+^CD4^+^ cells). J) Percentage of T_H_2 cells (CD3^+^CD4^+^IL‐4^+^, *n* = 5).

It is well recognized that M2 macrophages play a key role in tissue repair and regeneration, by exhibiting anti‐inflammatory and proregenerative characteristics, including clearance or inhibition of inflammatory cytokine transmission, awaking anti‐inflammatory response through releasing anti‐inflammatory cytokines and presenting T_H_2 immune response, and promoting angiogenesis. Therefore, the biologically functional activities of engineered artM were assessed in a study hypothesizing that artM might partially replicate the role of endogenous M2 macrophages during tissue regeneration. Firstly, monocytes and macrophages express a remarkable variety of receptor glycoproteins on their membranes, which involved in phagocytosis, metabolism, and activation of macrophages. Moreover, it is reported that the cell membrane of M2 macrophages have the ability to simultaneously absorb endotoxins and proinflammatory cytokines, contributing to manage sepsis caused by bacterial infection and orthotopic liver transplantation.^[^ ^15^ ^]^ Therefore, the engineered artM with macrophage membrane was expected to inherit several key biological characteristics of the native source cells. As shown in **Figure** **4**A, three key receptors were determined including CD14, a critical membrane protein responsible for LPS binding, CD120b, the cytokine‐binding receptor of tumor necrosis factor‐α (TNF‐α), and CD119, the cytokine‐binding receptor of interferon‐γ (IFN‐γ). The results demonstrated that these receptors were identified to be overexpressed in macrophage membrane and artM, indicating the inherent capability to specifically bind with proinflammatory mediators. Next, the ability of engineered artM in scavenging endotoxins and proinflammatory cytokines was further quantified through mixing artM with LPS and proinflammatory cytokines. As shown in Figure 4B–D, LPS and proinflammatory cytokines including TNF‐α and IFN‐γ can be effectively neutralized by artM in a concentration‐dependent manner, which was consistent with the previous reported macrophages membrane coated nanoparticles.^[^ ^16^ ^]^ Specifically, 4 mg mL^−1^ of artM could neutralize nearly 78.9% of LPS, 65.5% of TNF‐α and 98.3% of IFN‐γ after incubated at 37 °C for 30 min, respectively, indicating the preservation of membrane activity during artM fabrication. After the binding of artM to endotoxin and proinflammatory cytokines, it was reported that the complexes would be phagocytosed by monocytes, macrophages, and other phagocytic cells.^[^ ^17^ ^]^ Subsequently, these complexes are either transferred to other tissues or degraded intracellularly, which further contributes to the detoxification process. Therefore, this binding process was expected to block the interaction between the proinflammatory cytokines and their corresponding receptors on target cells, which would be beneficial for initially inhibiting the spread and deterioration of inflammation response in chronic wound microenvironment.

Next, the anti‐inflammatory bioactivity of artM was further assessed in macrophages polarization. BMDMs were used as reporter cells, and we firstly evaluated the effect of lysate proteins of M2 macrophages in BMDMs polarization. The results (Figure S3, Supporting Information) demonstrated that after incubation with the macrophage lysate, BMDMs displayed a shift into M2 phenotype with high expression of CD206, indicating the transition of M0 to M2‐type macrophages. Moreover, with the concentration of lysate increased, the proportion of M2 macrophages (F4/80^+^CD206^+^) was also increased, demonstrating a concentration‐dependent manner in macrophage M2 polarization induce by lysate proteins of M2 macrophages. Then, BMDMs were incubated with the engineered artM, and IL‐4 was selected as the positive control for inducing M2 macrophages polarization. As shown in Figure 4E,F, after gated with F4/80^+^ cells, the percentage of M2 macrophages was significantly elevated after artM treatment, while that of M1 macrophages (F4/80^+^CD86^+^) was not obviously influenced and maintained at a low level, suggesting artM could effectively mediate the polarization of macrophages toward M2 type. Macrophage polarization has also been found to elicit T‐cell response for mediating inflammatory response and tissue regeneration through cell crosstalk, which is evidence supporting the key role of T cells, including regulatory T cells, T_H_2, 𝛾𝛿T, and T_H_17 cells during wound repair.^[^ ^18^ ^]^ Herein, artM was incubated with lymphocytes in a co‐culture system to detect its function in provoking T_H_2 immune response (Figure 4H). The group containing lymphocytes alone served as the control group. After incubation for 48 h, flow cytometry was utilized to quantificationally verify the T_H_2 phenotype of lymphocytes. After initial gating on CD3^+^ and SSC‐A, and subsequential gating on CD4^+^, Figure 4I,J indicated a remarkable increase the proportion of T_H_2 (CD4^+^IL‐4^+^) cells (9.8 ± 3.8%) in T cells incubated with IL‐4‐induced M2 macrophages, while the proportion of T_H_2 cells maintained a low level in control, BMDMs and PLGA treatment groups. Significantly, artM treatment induced a comparable proportion of T_H_2 cells (9.4 ± 2.3%) compared with the M2 macrophages treatment, indicating the effective elicitation of T_H_2 response by artM. The expression level of *IL‐4* by activated lymphocytes was quantified by reverse transcription quantitative polymerase chain reaction (RT‐qPCR), which was associated with T_H_2 response. As shown in Figure S4A (Supporting Information), compared with the control group, the expression level of *IL‐4* was significantly elevated with the artM treatment, indicating the elicitation of Type 2/T_H_2/IL‐4 response. Increased expression of *IL‐4* can activate signal transduction of STAT6 and JAK1/3, which further improves the expression of T_H_2 cell characteristic transcription factor GATA‐binding protein 3 (GATA3), a key transcription factor for T_H_2 cell development.^[^ ^19^ ^]^ Therefore, we carried out RT‐qPCR to evaluate the signaling pathway of T_H_2 immune response activation. As shown in Figure S4B, compared with the control group, the expression level of *GATA3* was significantly upregulated after artM treatment, confirming the IL‐4‐dependent STAT6/GATA3 pathway in T_H_2 polarization. Altogether, it was suggested that the engineered artM could replicate the immunomodulatory function of endogenous M2 macrophages and exhibit unprecedented anti‐inflammatory activity including neutralizing proinflammatory mediators, inducing macrophages polarization into M2 type, and activating T_H_2 immune response via crosstalk with T cells.

### 2.3 Prohealing Activities of artM

**Figure 5:**
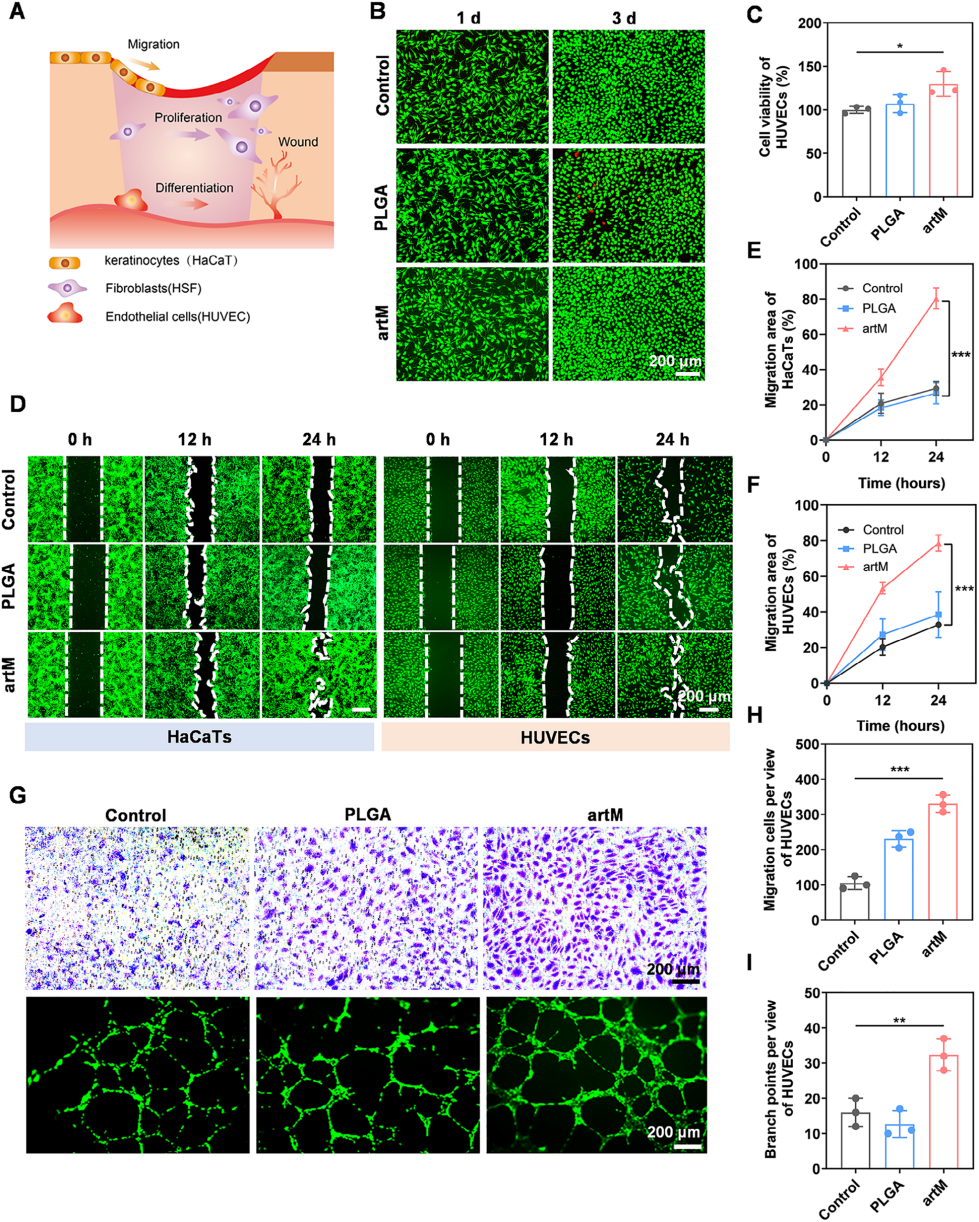
artM dedicated to wound healing by enhancing cell proliferation and migration, and angiogenesis. A) Illustration of the wound healing process involving the simultaneous participation of multiple cells. B) Live/dead assay of HUVECs after being incubated with PBS, PLGA and artM, respectively. C) The cell viability of HUVECs cultured with different formulations for 24 h determined by CCK‐8 kit (*n* = 3). D) Cell migration assay for HaCaT and HUVECs incubated with different formulations for 24 h. E,F) Quantitative analysis of cell migration area of HaCaT E) and HUVECs F) (*n* = 3). G,H) Representative images and quantitative analysis of transwell migration assay for HUVECs. G,I) Representative images and quantitative analysis of the tube formation of HUVECs treated with different formulations for 12 h (*n* = 3).

Wound healing process involves the simultaneous participation of multiple cells with diverse behaviors, such as fibroblast proliferation, keratinocyte migration, and endothelial cell differentiation (**Figure** **5**A), which is also regulated by macrophages. Thus, the effect of artM on the proliferation and migration of several cells including human umbilical vein endothelial cells (HUVECs), human immortalized keratinocytes (HaCaT), and fibroblasts (L929) was verified. First, the cytotoxicity of artM was verified through Cell Counting Kit‐8 (CCK‐8) assay. The results (Figure S5, Supporting Information) showed that there was no obvious cytotoxicity observed for L929 cells after treated with artM for 24 h at the concentration up to 50 µg mL^−1^, suggesting the cytocompatibility of artM. The hemolysis rates of artM at the tested concentration range of 1–100 µg mL^−1^ were lower than 0.5%, demonstrating an excellent hemocompatibility (Figure S6, Supporting Information). Then, artM with the concentration of 20 µg mL^−1^ were chosen for the following studies. L929 cells were further incubated with artM for 3 days and stained with Calcein‐AM/PI (Figure S7, Supporting Information). As shown in Figure 5B, numerous green fluorescence dots were observed, indicating that most cells were living and few cells died during cultivation. Moreover, compared with control and PLGA treatment, the number of cells with green fluorescence was significantly densely packed, demonstrating the continual proliferation of HUVECs with artM treatment. Quantification by CCK‐8 assay (Figure S8, Supporting Information; Figure 5C) suggested both L929 and HUVECs displayed significantly higher cell viability in artM treatment group, compared with other treatments, indicating that the engineered artM could support cell proliferation probably attributed to the presence of integrin on cell membrane and growth factors (e.g., VEGF) released from artM.

The re‐epithelialization process essential for wound closure requires keratinocytes and endothelial cells to migrate to the lesion site. Therefore, the scratch test was used to evaluate the effect of artM on the migration of HaCaT and HUVECs. Results in Figure 5D and Figure S9 (Supporting Information) demonstrated that both HaCaT and HUVECs in the artM group exhibited improved cell migration than that in the control group. The migration area of HaCaT and HUVECs was further counted, which also showed higher migration rate (Figure 5E,F) within 24 h in the artM group. In addition, transwell migration assay was also used to assess the migration of endothelial cells. Compared with the control group, more HUVECs were observed in artM group crossed the polycarbonate membrane, indicating that the artM effectively enhanced the mobility of endothelial cells (Figure 5G,H). The tube formation assay shown in Figure 5G,I and Figure S10 (Supporting Information) demonstrated HUVECs cultured with artM exhibited more junctions and higher tube density than the other groups, suggesting the superiority of artM in improving the tube formation of endothelial cells for promoting angiogenesis. Collectively, the engineered artM could enhance the cell proliferation of fibroblasts, migration of keratinocytes, and angiogenesis of endothelial cells, which would help to accelerate wound healing and tissue regeneration.

### 2.4 artM Accelerated Wound Healing of DTPI in Mice

Subsequently, in vivo degradation of artM was investigated through noninvasive fluorescence imaging. The artM labeled with Cy5 was subcutaneously injected into the back of mice and fluorescence images were recorded at the schedule time points. The results (Figure S11, Supporting Information) demonstrated that the fluorescence intensity was gradually decreased as a function of time, and about 11.7% of the initial artM remained after 14 days, indicating that artM was bioabsorbable in vivo.

**Figure 6:**
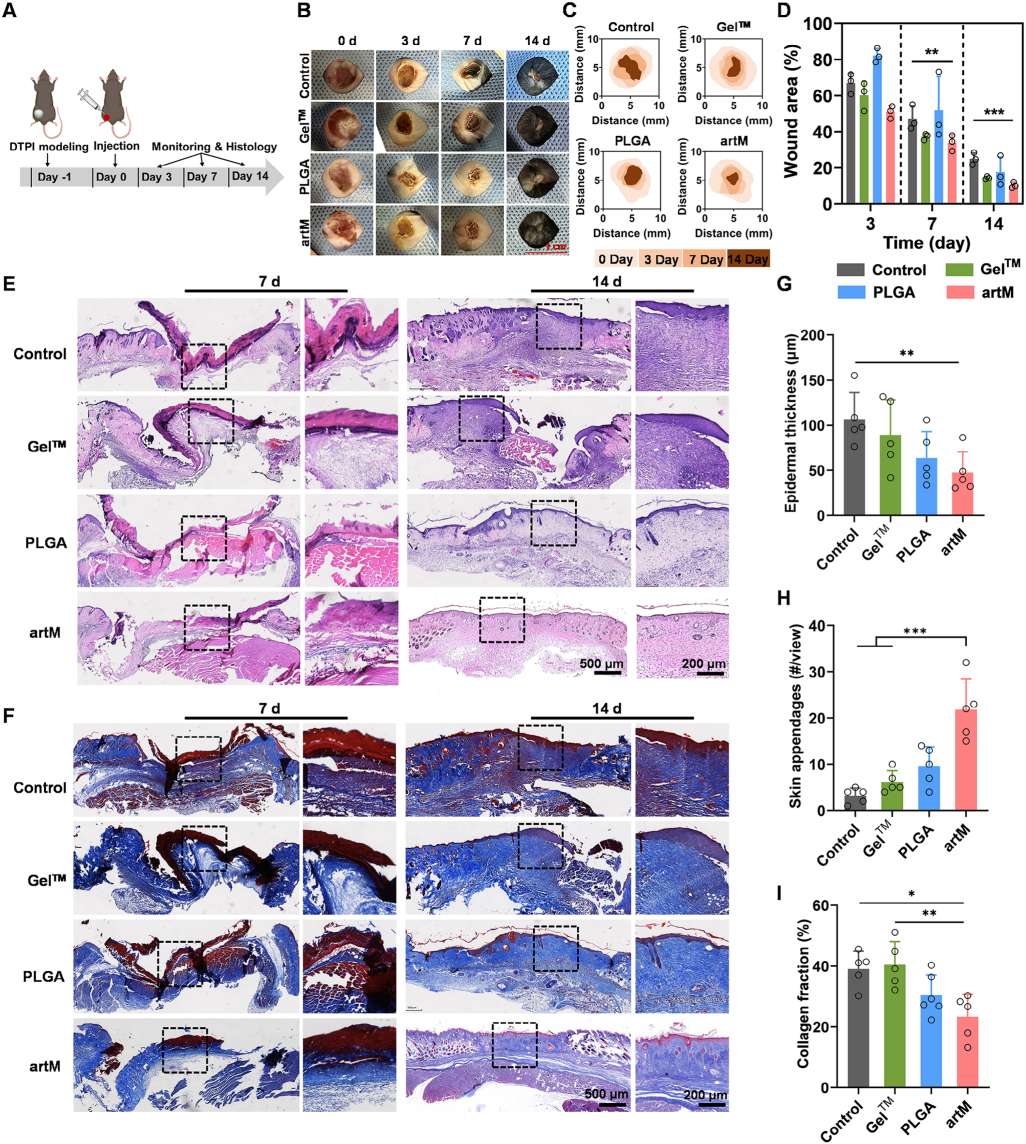
artM accelerated the wound healing and skin regeneration of DTPI in vivo. A) Schematic illustration of DTPI modeling and wound management procedure. B) Representative images of DTPI wounds treated with saline solution (as the control group), commercial Gel^TM^, PLGA microspheres and artM within 14 days. C) Schematic diagram of the dynamic wound healing process. D) Quantitative analysis of the wound area for each group (*n* = 3). E,F) Representative images of H&E staining E) and Masson's trichrome staining F) of the wounds at day 7 and day 14. G–I) Quantitative analysis of epidermal thickness G), skin appendage regeneration H) and collagen fraction I) at the wound site at day 14 (*n* = 5).

DTPI is a pressure‐induced local microcirculatory disturbance and repetitive ischemia/reperfusion injury, which is a typical nonhealing wound accompanied by recurrent and chronic inflammation.^[^ ^20^ ^]^ It has been reported that macrophage dysfunction dominated the destructive chronic inflammatory microenvironment of DTPI, and the accumulation of proinflammatory macrophages also inhibited the proliferation, migration, and differentiation of healing‐related cells.^[^ ^21^ ^]^ Encouraged by the superiority of engineered artM in anti‐inflammatory and proregenerative activities, we hypothesized artM would properly regulate the chronic inflammation and accelerate the tissue repair of skin DTPI. As shown in **Figure** **6**A, DTPI model in mice was established through constant pressure by magnets for 12 h. Epidermis and dermis layers of the skin were severely disrupted as indicated by hematoxylin and eosin (H&E) staining at day 0 (Figure S12, Supporting Information), corresponding to stage II to III according to the widely accepted NPIAP classification system in the United States. Then wound‐bearing mice were randomly grouped and received different treatments at the schedule time points. As recommend by the NPIAP system, commercially available chitosan hydrogel (Gel^TM^) was smeared on the wound every day as the positive control, since the wound was not infected and exudates were not obviously observed. Representative images of the wound were recorded and shown in Figure 6B, and the dynamic wound healing process was iconically displayed in Figure 6C according to a real‐time tracking. The results demonstrated that all skins under constant pressure exhibited obvious tissue necrosis and erythema, indicating the success of wound modeling. During the first few days, wounds in the control group progressed to severe ulcers with pus, demonstrating that wounds caused by DTPI was difficult to heal without intervention. Compared with the other groups, artM treatment significantly accelerated the wound healing, of which wounds were almost healed and covered by hair. The wound area at each time point were further analyzed and shown in Figure 6D. At day 7 post‐treatment, the wound area of artM treatment group (33.8 ± 4.3%) was lower than that of the control group (47.2 ± 6.9%). 14 days post treatment, artM‐treated wounds were nearly completely healed with a wound area of 10.3 ± 1.6%, which was significantly lower than that of the control group (24.9 ± 3.3%).

To further access the wound repair of the damaged skin, we conducted H&E and Masson's trichrome staining to evaluate the histopathological structure of the wound area. As shown in Figure 6E, at day 7, the epidermis in the control group displayed severe necrosis and pathological structure defect, and the dermis was destroyed without muscle or fat tissue, which was replaced by abundant collagen fibers. At day 14 post treatment, a few collagen fibers were observed in the wound, while a great number of immune cells and fibroblasts infiltrated in the wound bed in the control group. However, the pathological structures of skin were still incomplete with less regeneration of skin appendages. The treatment of commercial Gel^TM^ and PLGA slightly improved the outcome of wound regeneration. In contrast, artM treatment not only highly alleviated the epidermis and dermis defect at the early stage, but also accelerated the tissue repair, as indicated by more intact pathological structures and skin appendages regenerated in the wound bed after 14 days. Moreover, Masson's trichrome staining also demonstrated that the wounds with artM treatment showed few but well‐organized collagen fibers, in comparison with that of the control group at day 14 (Figure 6F). The epidermal thickness and the number of new‐born skin appendages in the repaired tissue were counted (Figure 6G,H). Compared with the other treatment groups, artM treatment showed significant decrease in epidermal thickness and an apparently larger number of regenerated skin appendages, indicating that the engineered artM potently triggered the regeneration of an intact epidermis layer and a mature dermis. Additionally, well‐organized deposition and bundles of collagen fibers are necessary during the remodeling phase of wound healing. As shown in Figure 6I, the fraction of collagen area calculated from Masson's staining demonstrated that artM notably reduced the collagen density, compared with control and Gel^TM^ treatment, which may be conducive to scarless healing. These results indicated that the artM greatly promoted the wound healing of skin DTPI, including the regeneration of epidermis, dermis and skin appendages, and the proper deposition of collagens. Hence, the use of artM could prevent the transition of ulcer from low stage to a higher grade, offering a new management modality. To evaluate the biocompatibility of artM, we subcutaneously injected artM into mice and harvested major organs (e.g., heart, liver, spleen, lung, and kidney) 14 days post‐injection for histological analysis. H&E staining revealed no significant signs of inflammation or tissue damage in the examined organs, confirming the biosafety of artM (Figure S13, Supporting Information).

### 2.5 artM Modulated the Inflammation and Promoted Angiogenesis in Wound Site

**Figure 7:**
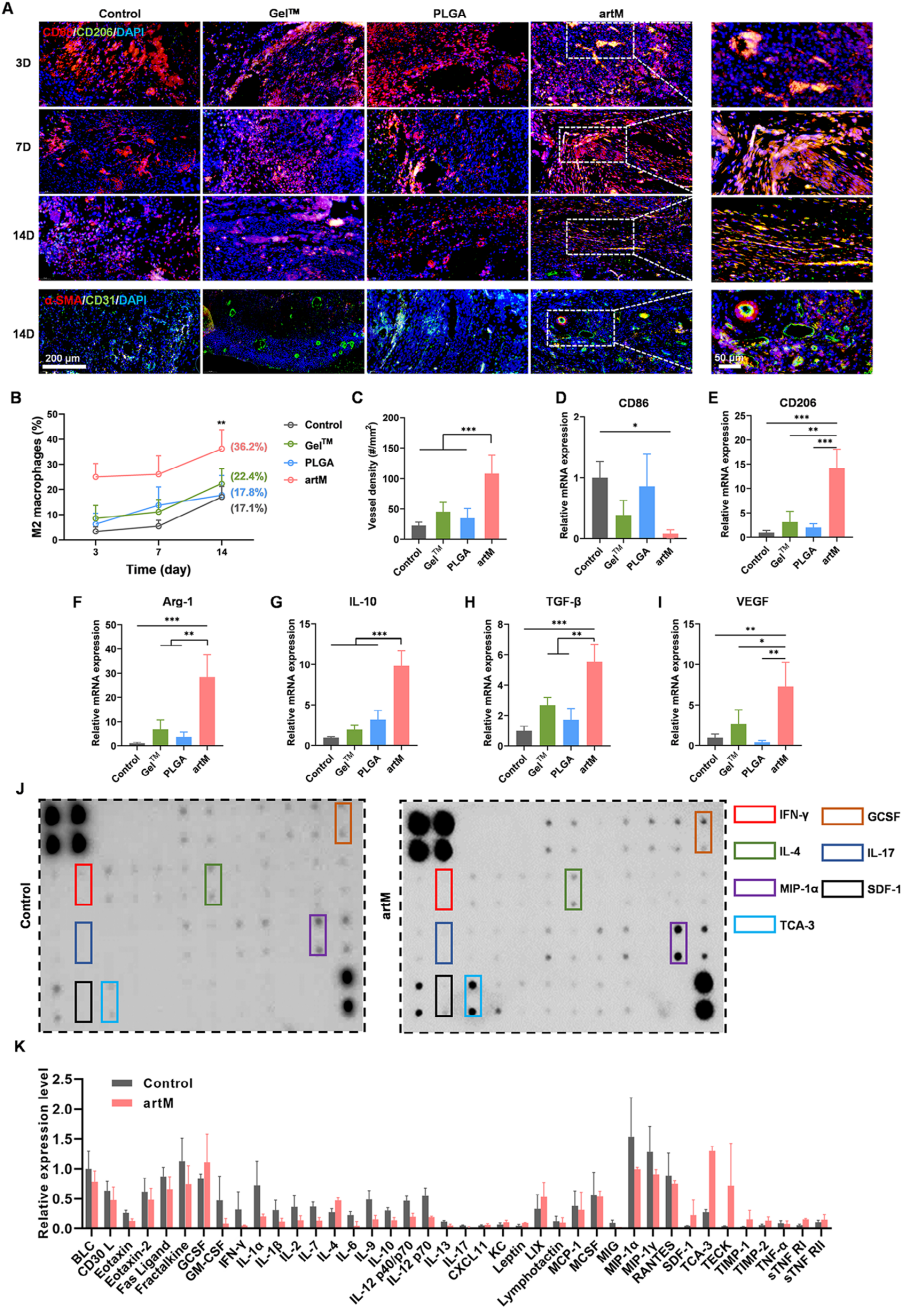
artM facilitated wound healing via regulating inflammation response and promoting angiogenesis. A) Representative images of CD68/CD206 and CD31/α‐SMA immunofluorescence staining at the wound site. B) Quantification of M2 macrophages population at the wound site during the wound healing process (*n* = 5). C) Quantification of vessel density at the wound site according to the CD31/α‐SMA immunofluorescence staining at day 14 (*n* = 5). D–I) Relative mRNA expression of *CD86*, *CD206*, *Arg‐1*, *IL‐10*, *TGF‐β* and *VEGF* in skin wound at day 7 post treatment (*n* = 3). J, K) Cytokine array assay and quantitative analysis of the proinflammatory cytokine level in the wounds at day 7 post treatment (*n* = 3).

To verify the mechanism of artM in expediting wound healing after DTPI, we attempted to access the activity in inflammation modulation, since dysregulated immune balance is a fundamental pathological factor underlying the nonhealing state of DTPI. We first performed the immunofluorescence staining to detect the macrophages infiltration at wound site during the treatment. CD206 was chosen as a surface marker for M2 macrophages, and CD68 was used to identify macrophages of all subsets. As shown in **Figure** **7**A, at day 3, a widespread distribution of CD68^+^ cells (red fluorescence) were found in all groups, especially in control group, indicating a high level of inflammation response in skin with DTPI at the early stage. Compared with other treatments, there were more CD68^+^CD206^+^ cells (yellow fluorescence) in the subcutaneous layer of wound in artM treatment group during the whole observation period. Moreover, the percentages of M2 macrophages in artM treatment group were higher than these in other groups and continually increased from 3 to 14 days (Figure 7B). Then, macrophages in the wound tissues on day 14 were further quantitatively analyzed by flow cytometry. The results (Figure S14, Supporting Information) demonstrated that, compared to the control, Gel^TM^, and PLGA groups, the artM group exhibited a significantly higher proportion of M2 macrophages (32.9 ± 5.5%) and a significantly lower proportion of M1 macrophages (6.2 ± 1.2%). These findings indicate that artM effectively modulates macrophage polarization in vivo, promoting a shift toward the anti‐inflammatory M2 phenotype. The vasculature in DTPI is largely defected due to compromised angiogenesis and deformed capillary network, both of which are related to macrophage dysfunction. Therefore, we further interested in whether artM could restore the vasculature in skin DTPI. CD31 is a transmembrane protein expressed in early angiogenesis, demonstrating neovascularization, while 𝛼‐SMA is a cytoplasmic protein expressed in later angiogenesis, indicating the maturation of vascular smooth muscle cells.^[^ ^22^ ^]^ As shown in Figure 7A,C, artM treatment induced a higher expression of CD31 and 𝛼‐SMA proteins and resulted in a higher density of vessels in dermis in the wound bed, compared with that of the other groups. The angiogenesis in artM group was mainly attributed to the elevated M2 macrophages in wound site and the release of growth factors from the engineered artM. Then, to further identify the immune regulation effect of artM from the molecular biology level, the expression of mRNA encoding factors associated with M2 macrophages and angiogenesis were evaluated by RT‐qPCR. As shown in Figure 7D–I, artM treatment significantly elevated the expression of M2 macrophages associated mRNA, including *CD206*, *Arg‐1*, *IL‐10*, and transforming growth factor‐β (*TGF‐β*), and downregulated the expression of M1 associated mRNA (*CD86*). Furthermore, compared with other treatments, the expression of VEGF was also significantly upregulated with artM treatment. Proinflammatory cytokine expression in the wound site was assessed by cytokine array analysis. As shown in Figure 7J,K, compared with the control group, artM treatment reduced the expression of various proinflammatory cytokines, including IFN‐γ, macrophage inflammatory protein‐1α (MIP‐1α), IL‐17 and IL‐6, while up‐regulated the expression of anti‐inflammatory cytokines, such as T cell activation gene‐3 (TCA‐3), stromal cell derived factor‐1 (SDF‐1), IL‐4 and granulocyte colony‐stimulating factor (G‐CSF). We have further validated the levels of IL‐10 in the regenerated tissue using ELISA. The results demonstrated that the artM‐treated group exhibited a significant increase in IL‐10 levels compared to the control, Gel^TM^, and PLGA groups (Figure S16, Supporting Information). These findings suggested artM could effectively dampen the local inflammatory response by driving M2 type macrophage polarization, diminishing the excessive secretion of proinflammatory cytokines, and promoting angiogenesis, thus re‐shaping the regenerative microenvironment for the healing of DTPI.

### 2.6 artM Accelerated Wound Healing of DTPI with Macrophages Depletion In vivo

**Figure 8:**
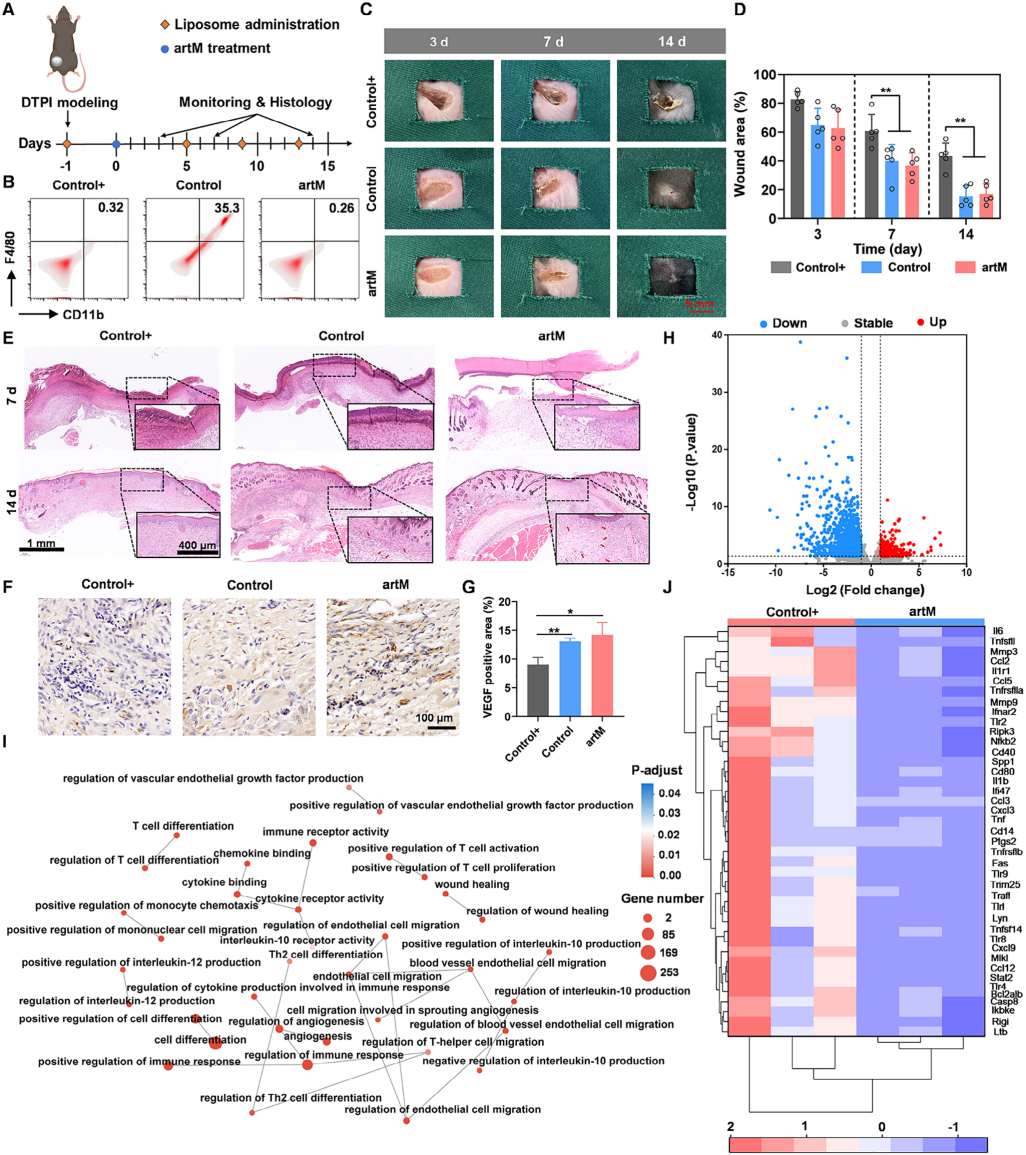
artM expedited the tissue regeneration of DTPI in macrophage depletion model. A) Schematic illustration of macrophage depletion and wound management procedure. B) Flow cytometry analysis of CD11b and F4/80 expression of cells harvested from the wound site. C) Representative images of DTPI wounds with macrophages depletion (Control+ group), without macrophages depletion (Control group) and artM treatment with macrophages depletion within 14 days. D) Quantitative analysis of wound area for each group (*n* = 5). E) Representative images of H&E staining of the harvested wounds at day 7 and day 14. F,G) Immunohistochemical staining F) and quantification of VEGF (G) (*n* = 3). H) Volcano map of gene expression in artM treatment group compared with the Control+ group. Red points and blue points represent upregulated and downregulated genes based on *p*‐value *<* 0.05 and |logFC| *>* 1, while gray points represent genes with no significant difference. I) GO enrichment analysis of DEGs. J) Cluster heatmap of the top 41 upregulated and downregulated DEGs. DEGs, differentially expressed genes.

To further verify the role of artM in immune regulation and proregenerative function as a living macrophage cell substitute for wound healing, macrophages depletion model was constructed using C57BL/6 mice through intraperitoneal administration of clodronate liposomes during the whole experimental period (**Figure** **8**A). Clodronate liposomes could deplete local macrophages and prevent new macrophages and monocytes migration toward the wound region. Wounds with macrophage depletion were adopted as positive controls (termed as Control+). As shown in Figure 8B, the wounds were harvested and the populations of macrophages were analyzed by flow cytometry. The results demonstrated that compared with the Control group, the clodronate liposomes treatment could effectively deplete macrophages at the wound site. Subsequently, DTPI models were established in mice received with macrophage depletion and treated with artM to evaluate its therapeutic effects on wound healing. The representative images of skin wounds at different time points were shown in Figure 8C. An obviously delayed healing rate in Control+ group was found, in comparison with the other groups, indicating that macrophages were necessary for wound healing. However, the administration of artM significantly accelerated the wound healing process, which was similar with the Control group. Quantitative analysis of wound area in Figure 8D also confirmed the wound closure rate of artM treatment in macrophage depletion model was almost the same as that in the Control group, which was significantly higher than that of the Control+ group. These outcomes demonstrated the engineered artM could play the functional role of macrophages in tissue regeneration.

H&E staining toward histopathological structure of skin (Figure 8E) revealed severe necrosis and pathological structure defect in the wounds with macrophage clearance at day 7, and there was still large wound area with incomplete skin pathological structures and granulation tissue was almost absent at day 14. In comparison, the wounds in Control and artM groups displayed a faster wound closure, and smaller wound area and intact epidermis and dermis layers could be observed with renascent granulation tissues (indicated by red arrows) at day 14. Further, the number of skin appendages and epidermal thickness were quantitatively analyzed in the wound area. Both artM treatment and Control group exhibited a greater number of newly regenerated appendages and a thinner epidermis (Figure S16, Supporting Information), compared with the Control+ group, suggesting a significantly better wound healing outcome. Reduced angiogenesis plays a prominent negative role in the nonhealing nature of chronic wounds.^[^ ^23^ ^]^ VEGF is important in reconstructing the vascular network, which is deficient in chronic wounds.^[^ ^24^ ^]^ As shown in Figure 8F,G, the level of VEGF in artM‐treated wounds was significantly higher than in wounds with macrophage depletion.

To further investigate the mechanism of artM as a substitute for endogenous macrophages in DTPI therapy, we proceeded with transcriptome sequencing (RNA‐seq) of the skin tissues from both the artM treatment group and the Control+ group. Based on the predefined criteria (*p*‐value < 0.05 and |logFC| > 1), a total of 1919 DEGs were identified. Among them, the expression of 369 genes was upregulated, while 1550 genes were downregulated. These DEGs were visually represented through volcano plots (Figure 8H). Furthermore, the GO enrichment analysis was used to categorize the functions of these DEGs. As shown in Figure 8I, the DEGs were mainly associated with wound healing, positive regulation of endothelial cell migration, regulation of immune response, and angiogenesis, contributing to immunomodulatory and proregenerative functions of artM. Kyoto Encyclopedia of Genes and Genomes (KEGG) pathway enrichment analysis was presented in Figure S17 (Supporting Information). Notably, significantly enriched terms within KEGG encompassed cytokine–cytokine receptor interaction and inflammation‐related signaling pathways, thereby indicating that artM could alleviate inflammation through modulating the activation of immune cells. Subsequently, Gene Set Enrichment Analysis (GSEA) demonstrated the DEGs were enriched within “Toll‐like receptor signaling pathway”, “TNF signaling pathway”, and “Chemokine signaling pathway” with a down‐regulation character (Figure S18, Supporting Information), which suggested the resolution of inflammation. Meanwhile, the genes corresponding to the pathway of interest (KEGG) shown in the cluster heatmap highlighted the evident decrease in gene expression of inflammation‐related cytokines, such as IL‐6 and TNF, and ECM‐degrading protein enzymes, such as matrix metalloproteinase‐3 (MMP‐3) and MMP‐9 in artM treatment group (Figure 8J). In addition, the PPI network was constructed for the DEGs to analyze the interactions between the proteins.^[^ ^25^ ^]^ The network formed by the top 25 highly related genes (Figure S19, Supporting Information) demonstrated the function of these gene encoded proteins was related to inflammation and angiogenesis. These data substantiated that the artM augmented the treatment of skin ulcer by regulating the inflammatory microenvironment, and promoting angiogenesis.

## 3 Discussion

Our findings demonstrated that the artM was able to effectively replicate the biological function of endogenous prorepair macrophages, promoting the healing and regeneration of chronic skin injury. In the context of regenerative cell engineering, the artM represents an alternative approach capable of addressing the primary limitations of cell therapies using live macrophages modified by *ex vivo* or in vivo engineering technology. A central innovation lies in the encapsulation of macrophage lysate within biodegradable PLGA microspheres, enabling sustained and controlled release of bioactive factors (e.g., VEGF and IL‐10). Negligible protein leakage was observed during the fabrication of lysate‐loaded microspheres, demonstrating the high encapsulation efficiency of the engineered delivery system. Stability assessments validated the preservation of therapeutic efficacy in artM following 28‐day cryopreservation at −80 °C, offering a distinct advantage over conventional cell therapies that suffer from rapid decline of viability and functionality under suboptimal storage conditions. The cryo‐stability and scalability align with the emergence of cell‐free regenerative strategies, facilitating its translation into clinical workflows. Furthermore, artM, containing multiple cytokines and growth factors, may hold therapeutic superiority over single‐factor delivery systems by replicating the multifactorial paracrine signaling cascade inherent to native macrophages.

The artM platform incorporates functional macrophage membrane, which not only preserves the native targeting specificity, but also maintains various critical surface receptors, such as CD14, CD120b, and CD119, which are involved in responses to LPS, TNF‐α, and IFN‐γ, respectively. Cytokine neutralization assays demonstrated the significant clearance efficiency against inflammatory mediators, effectively mitigating the proinflammatory microenvironment of pressure‐induced chronic wound.^[^ ^26^ ^]^ By mediating the sequestration of proinflammatory molecules and promoting the sustained release of anti‐inflammatory cytokines, this process effectively breaks the self‐perpetuating cycle of inflammation and hindered healing.

Regarding the interaction of artM with stromal or immune cells, our results confirmed that artM enhanced fibroblast proliferation, keratinocyte migration, and endothelial cell migration. The interaction between artM and cells is primarily driven by the release of cytokines or growth factors due to the nonliving nature of artM. VEGF has been shown to be sustainably released from artM. When in contact with endothelial cells, VEGF binds to VEGFR2, triggering the downstream PLCγ‐PKC‐ERK1/2 and FAK signaling pathways, thereby promoting cell proliferation, migration, and vascular homeostasis.^[^ ^27^ ^]^ In addition, artM present at the wound site could also act as an endogenous tissue engineering scaffold, offering a three‐dimensional extracellular matrix substitute that supports cell infiltration, adhesion, and proliferation. Notably, artM could initiate a cascade of reparative response via polarizing naïve T‐cell differentiation toward anti‐inflammatory T_H_2 phenotypes through IL‐4‐dependent STAT6/GATA3 activation, thereby mimicking the immunomodulatory functions of natural macrophages. Besides, IL‐10 loaded in artM was also able to induce T_H_2 polarization.^[^ ^28^ ^]^ Vice versa, T_H_2 cells can secrete IL‐4 and IL‐10 to prime endogenous macrophage into M2 phenotype.^[^ ^29^ ^]^ Although these preliminary findings establish the immunomodulatory potential of artM in fostering proregenerative immune responses and curtailing excessive inflammation, it remains to be determined whether this platform also suppresses proinflammatory T‐cell subsets (e.g., T_H_1, T_H_17) within the damaged tissue from the perspective of cell crosstalk.

Despite the results obtained, this work has several limitations. To begin with, as a nonliving system, artM could not fully replicate biological behaviors of macrophages, such as proliferation, differentiation, and phagocytosis. Therefore, a thorough evaluation of its superiority over the adoptive transfer of living engineered macrophages is required. Only through this can we truly determine whether artM has the potential to replace live macrophages in regenerative medicine. In addition, to further ensure inter‐batch consistency and scalability, advanced engineering strategies, such as magnetic‐activated cell sorting for macrophage subpopulation purification or microfluidic‐based production, can be employed. These limitations further emphasize the need for additional considerations in the design of artificial cellular systems intended to replicate the intricate immune modulation during wound healing.

Despite these challenges, artM presents significant translational potential for the treatment of chronic inflammatory conditions and tissue repair, especially in cases of hard‐to‐heal skin wounds. Its off‐the‐shelf applicability, high‐efficiency in wound healing, and exceptional stability could significantly facilitate clinical translation. The platform exemplifies a broader shift toward hybrid bioengineering, where synthetic materials and biological elements (including proteins, organelles, membranes, and cells) are integrated to tackle unresolved medical challenges. Through the integration of materials, engineering, and biology, artM advances the paradigm of cell‐mimetic therapeutics, emphasizing stability, reproducibility, and multifunctional bioactivity as critical benchmarks for next‐generation regenerative medicine.

## 4 Conclusion

Here, the artM was constructed as an off‐the‐shelf product that could maintain the phenotype and function of living macrophages and contribute to tissue repair after DTPI. The artM was successfully assembled by biodegradable PLGA microspheres loaded with lysate proteins derived from M2 macrophages, which was further coated with macrophage membrane on the microspheres surface. It was proven artM could be stored in culture medium‐free, low‐temperature environment with long‐term stability. More importantly, artM could act like autologous prohealing macrophages after transfusion in vivo, and guide the repair of DTPI‐induced skin ulcer via coordinating immune regulation, angiogenesis, and tissue remodeling. Wound healing on macrophage depletion models further confirmed the efficacy of artM in facilitating endogenous tissue regeneration. Such an engineered artM may serve as an acellular and clinically feasible alternative in the prevention and treatment of DTPI. It is envisioned the immunomodulatory and prorestorative artM may hold potential not only in wound care, such as diabetic ulcer and myocardial infarction, but also in treating inflammatory disease, including myocarditis and enteritis.

## 5 Experimental Section

### Materials and Animals

Poly (d,l‐lactide‐co‐glycolide) (50:50, molecular weight 30000 Da), polyvinyl alcohol (PVA) and dichloromethane were purchased from Sigma‐Aldrich (St. Louis, MO, USA). Information on the other chemical and biological reagents was listed in the Supporting Information. C57BL/6 (male/female, 6–8 weeks) were purchased from Huafukang Bioscience Co., Ltd. (Beijing, China). All animal procedures were performed in accordance with the rules established by the Animal Experiment Ethics Committee and Authority of Institute of Radiation Medicine, Chinese Academy of Medical Sciences (Approval No: SYXK (Jin) 2019‐0002).

### Isolation of Lysate Protein and Membrane from M2‐type Macrophages

BMDMs were isolated from C57BL/6 mice (6 weeks old, female) according to the previous report.^[^ ^12a^ ^]^ Briefly, bone marrow of femurs and tibia was first flushed with PBS. After lysing red blood cells, the collected cells were seeded in 6 well plate and cultured with RPMI‐1640 medium supplemented with 10% FBS (Gibco, Grand Island, NY, USA) and 20 ng mL^−1^ M‐CSF. After incubation for 6 days in an incubator (37 °C, 5% CO_2_), most adhered cells were BMDMs. BMDMs were seeded in a 6‐well plate at the density of 4 × 10^5^ cells per well. Then, in order to induce the polarization of macrophages to M2‐type, IL‐4 (40 ng mL^−1^) was added and further incubated for 48 h. To evaluate the polarization effect of macrophages, the cells were collected and stained with CD86, F4/80 and CD206 antibodies, which were analyzed by flow cytometry (C6, BD Biosciences, San Jose, CA, USA). To observe the cell morphology, BMDMs were seeded in confocal dishes, treated with IL‐4 for 48 h, and then stained with FITC‐conjugated phalloidin, DAPI and CD206 antibodies. The images were obtained by CLSM (TCS SP5II, Leica, Ernst‐Leitz‐Strasse, Germany).

IL‐4 induced M2 macrophages (5 × 10^7^) were collected and washed twice with cold PBS. After that, the cells were resuspended in membrane protein extraction reagent A plus 1 mM PMSF (ST506, Beyotime, Shanghai, China) and completely dissolved at 4 °C for 15 min. Proteins of M2 macrophage lysate were obtained through repeated freezing and thawing in liquid nitrogen for 3 times, and the supernatant was collected by centrifugation for further purifying by Membrane and Cytosol Protein Extraction Kit (P0033, Beyotime, Shanghai, China) according to the standard protocols. The lysate proteins from M2‐type macrophages were determined by proteomic sequencing (Majorbio BioTech Co., Ltd., Shanghai, China). Subsequently, to isolate the cell membrane, treated cells were harvested and suspended in a homogenization buffer containing 20 × 10^−3^ m of Tris·HCl (pH 7.5), 10 × 10^−3^ m of KCl, 2 × 10^−3^ m of MgCl_2_, 75 × 10^−3^ m of sucrose, and a protease/phosphatase inhibitor tablet. After disrupting the suspension by ultrasound (ultrasonic power:100 W; duration: 10 s pulses with 10 s intervals) at 4 °C for 10 min, the suspension was subjected to centrifugation at 20 000 × *g* for 25 min. The resulting supernatant was further centrifuged at 100 000 × *g* for 35 min to collect the cell membrane, which was then stored at −80 °C. The content of the membrane was quantified with BSA kit (P0398S, Beyotime, Shanghai, China).

### Preparation and Characterization of artM

PLGA‐Lys were fabricated by a water‐oil‐water (w/o/w) emulsion technique. 10 mg of lysate proteins were dissolved in PVA solution (0.1 wt%) as the internal aqueous phase, and 3 mL dichloromethane containing 100 mg PLGA was used as the oil phase. The mixed solution was emulsified by ultrasound (ultrasonic power: 100 W; duration: 10 s pulses with 10 s intervals) in an ice bath at 4 °C for 10 min. Next, the emulsion was poured into a rapidly stirred PVA solution (30 mL, 1 wt%) at 4000 rpm for 10 min, and the mixture was stirred at 25 °C for 4 h to evaporate the solvent. Finally, the PLGA‐Lys were centrifuged for 5 min at 3000 rpm, washed with deionized water 3 times and resuspended in PBS for further use. To prepare PLGA microspheres as a control, the same amount of deionized water was added to the PLGA solution and the other steps were the same as above. The morphology, size, and zeta potential of PLGA and PLGA‐Lys microspheres were analyzed by SEM (Regulus 8100, Hitachi, Tokyo, Japan) and Zetasizer Nano ZS (Malvern, UK), respectively. To evaluate the encapsulation efficiency of lysate within PLGA microspheres, 1 mg of lysate proteins was encapsulated during the preparation of PLGA microspheres using 100 mg of PLGA polymer. After the synthesis of PLGA‐Lys, the sample was washed three times with PBS and then centrifuged at 3000 rpm for 10 min to separate unencapsulated proteins. The supernatant was subsequently collected and analyzed for free protein concentration using a BCA assay kit according to the manufacturer's protocol. And the encapsulation efficiency was calculated by the formulation:

(1) $$\mathrm{Encapsulation}\;\mathrm{efficiency}\;\left(\%\right)=\left(1-\frac{\mathrm{Protein}\;\mathrm{in}\;\mathrm{the}\;\mathrm{supernatant}}{\mathrm{Original}\;\mathrm{protein}}\right)\times 100$$

Subsequently, the isolated cell membrane of M2 macrophages was mixed with PLGA‐Lys and treated by ultrasound (100 W) for 2 min. The suspension was extruded using a polycarbonate membrane to prepare artM. TEM (JEM‐1011, JEOL, Tokyo, Japan) was used to verify the microstructure of PLGA‐Lys. XPS (Thermo Scientific K‐Alpha, USA) was employed to detect the chemical composition of artM. To observe the structure and composition of artM, 1,1′‐dioctadecyl‐3,3,3′,3′‐tetramethylindocarbocyanine perchlorate (DiI, C1991S, Beyotime, Shanghai, China), Fluorescein isothiocyanate (FITC, MCE) and Sulfo‐Cyanine5 (Cy5, MCE) were used to label the cell membrane, lysate and PLGA, respectively, and photographed with CLSM. To detect the stability, 0.1 mg mL^−1^ of artM was incubated in PBS containing 10% FBS, and the particle size was determined by DLS at scheduled time.

### Cryostability Analysis of artM

To explore the cryostability of artM, we generated the first batch of artM (*n* = 4), which was stored at −80 °C. On day 28, the first batch was thawed and the second batch of artM was freshly prepared. All batches of artM were rinsed three times with sterile PBS and then resuspended in 1 mL sterile PBS. Next, artM was incubated for 24 h in an incubator at 37 °C. Cytokines (IL‐10 and VEGF) in the supernatant were quantified by ELISA kit (CUSABIO, Wuhan, China).

### Cell Proliferation, Migration, and Tube Formation Assays

The cell viability was tested by live/dead assay kit (C2015 M, Beyotime, Shanghai, China). Subsequently, the wound scratch assay was carried out on both HUVECs and HaCat cells. Additionally, the transwell migration and tube formation assays were performed on HUVECs. The details were described in the Supporting Information.

### Endotoxin and Cytokine Neutralization

To evaluate the neutralization capacity of artM, LPS (600 ng) was incubated with artM (1 or 4 mg mL^−1^) for 30 min. The supernatant was collected by centrifugation (16 000 × *g*, 20 min), and the concentration of free LPS was quantified using LPS ELISA (Cloud‐clone, Wuhan, China). To assess the binding capability to proinflammatory cytokines (TNF‐α, IFN‐γ) of artM, samples were mixed with TNF‐α and IFN‐γ, incubated for 30 min. After incubation, cytokine levels in the supernatant were quantified by ELISA (Elabscience, Wuhan, China).

### In Vitro Macrophage Polarization Assay

BMDMs were treated with PBS, IL‐4 (40 ng mL^−1^), PLGA, PLGA‐Lys (20 µg mL^−1^), or artM (20 µg mL^−1^) for 48 h. Then, BMDMs were stained with antibodies and analyzed by flow cytometry. Detailed protocols are provided in the Supporting Information.

### Crosstalk of Macrophages and T cells

Spleen lymphocytes were isolated from the spleen of C57BL/6 mice using a lymphocyte isolation reagent (Solarbio, Beijing, China). The spleen lymphocytes were cocultured with PBS, BMDMs, M2 macrophages (IL‐4‐treated BMDMs), PLGA, or artM for 48 h. Then the lymphocytes were stained with antibodies and analyzed by flow cytometry. Detailed protocols are described in the Supporting Information.

### Deep Tissue Pressure Injury Mouse (DTPI) Model

C57BL/6 mice (male, 8–10 weeks, 20–22 g) were used to establish the DTPI model. The dorsal fur was removed using electric clippers followed by a depilatory cream. The skin was disinfected with 70% ethanol and iodine. Mice were anesthetized via intraperitoneal injection of 2% pentobarbital sodium (45 mg k^−1^g). Two disc‐shaped neodymium magnets (12 mm in diameter, 5 mm in thickness, 2.4 g, 1000 Gauss) were placed on the dorsal and ventral skin overlying the ischial spine, respectively. Pressure was applied for 12 h to induce ischemia, followed by 12 h of reperfusion. One cycle was completed for model establishment. Each mouse was kept in a single cage during the 12 h stress period and was allowed to eat and water freely. Then the mice were randomly divided into four groups and subcutaneously injected with 100 µL of sterile saline solution, chitosan hydrogel (Gel^TM^, Humanwell Pharmaceutical Group Medical Products Co., Ltd. China), PLGA (2 mg mL^−1^) or artM (2 mg mL^−1^), respectively. The wounds were photographed at 0, 3, 7, and 14 days after injection. Wound areas were quantified using ImageJ. On day 7 and 14, mice were euthanized, and skin tissues were processed for H&E staining and Masson's trichrome staining.

### Cytokine Array Analysis

Cytokine array analysis was used to assess the expression of proinflammatory cytokines at the wound site. Detailed experimental procedures are described in the Supporting Information.

### Macrophage Depletion in vivo via Clodronate Administration

To further evaluate the function of artM, mice with DTPI model were randomly divided into three groups. Group 1 (Control): Mice received subcutaneous injections of 0.9% saline without macrophage depletion. Group 2 (Control+): Mice received intraperitoneal clodronate liposomes (50 mg k^−1^g, cat# CP‐005‐005, LIPOSOMA B.V., Netherlands) every 3 days. Group 3 (artM): Mice were treated with clodronate liposomes and subcutaneously injected with artM (100 µL) once. Macrophage depletion was verified by F4/80 and CD11b staining. Mice were sacrificed on day 7 and 14, and skin tissues were harvested, fixed in 4% paraformaldehyde, and processed for H&E staining and immunohistochemistry. Detailed protocols for immunofluorescence, flow cytometry, and genomic analysis are provided in the Supporting Information.

### Statistical Analysis

Data were presented as mean ± standard deviation (SD). Differences between two groups were analyzed using Student's t‐test, while comparisons among multiple groups were assessed by one‐way ANOVA (GraphPad Prism 9). Statistical significance is denoted as follows: *p < 0.05, **p < 0.01, and ***p < 0.001.

## Conflict of Interest

The Authors declare no conflict of interest.

## Supporting information



Supporting Information

## Data Availability

All data are available in the main text or the supplementary materials.
