# Supplementary material for: An Off‐the‐Shelf Artificial Proregenerative Macrophage for Pressure Ulcer Treatment
Source: Adv Sci (Weinh). 2025 Apr 24;12(21):2415886. doi: 10.1002/advs.202415886 (PMC12140330; doi:10.1002/advs.202415886)
Supplement: Supplementary file 1 — Supporting Information [file ADVS-12-2415886-s001.docx]

**Supporting Information**

**An Off-the-Shelf Artificial Pro-Regenerative Macrophage for Pressure Ulcer Treatment**

*Qi Su, Jingrong Wang, Yini Huangfu, Rui Gao, Pengxu Kong, Yu Gao, Huijuan Song*, Ju Zhang, Pingsheng Huang, Chuangnian Zhang, Zujian Feng*, Deling Kong, Weiwei Wang**

***Materials:*** Recombinant mouse M-CSF and IL-4 were purchased from PeproTech (Rocky Hill, NJ, USA). Fluorochrome- conjugated monoclonal antibodies (CD3, CD4, IL-4, F4/80, CD86 and CD206) were provided by BioLegend (San Diego, CA, USA). Primary antibodies (CD14, CD120b, CD68, CD206, CD31, α-SMA) were purchased from Abcam (Cambridge, UK). HiScript III Reverse Transcriptase and AceQ Universal SYBR qPCR Master Mix were purchased from Vazyme Biotech (Nanjing, China). RAW264.7 cells, L929, HUVECs and HaCaT cells were purchased from Procell Life Science & Technology Pricella (Wuhan, China) and cultured per the manufacturer’s protocols. Cell counting kit-8 assay kit (CCK-8, CA1201) and Total RNA Extraction Kit (R1200) were purchased from Solarbio (Beijing, China). All medium required for cell culture were purchased from Thermo Fisher Scientific (Waltham, MA, USA). ELISA kits (IL-10 and VEGF) were provided by CUSABIO (Wuhan, China).

***Encapsulation Efficiency Determination:*** Following the synthesis of PLGA-Lys, the samples were subjected to centrifugation at 3,000 rpm for a duration of 10 min to separate the supernatant. Free protein concentration in the supernatant was quantified using a BCA assay kit (Solarbio, Cat. No. PC0020, Beijing, China), with absorbance measured at 562 nm.

***Cytocompatibility Evaluation of artM:*** Cell viability was evaluated by CCK-8. Briefly, L929 cells were seeded at a density of 1 × 10^4^ cells/well in a 96-well plate and were treated with artM (0, 1, 2, 10, 20, or 50 μg/mL) for 24 h. Cell viability was assessed using a CCK-8 assay kit according to the manufacturer’s protocol. The absorbance was measured at 450 nm.

***Hemolysis Assay:*** Briefly, fresh rat erythrocytes were isolated from whole blood by centrifugation (1500 × g, 10 min) and washed three times with phosphate-buffered saline (PBS). The erythrocytes were then resuspended in PBS to prepare a 2% (v/v) suspension. Different concentrations of artM (1, 5, 10, 50, and 100 μg/mL) were incubated with the erythrocyte suspension at 37 °C for 1 h. Saline (0.9% NaCl) and deionized water served as the negative control (0.1% hemolysis) and positive control (99.5% hemolysis), respectively. After incubation, the mixtures were centrifuged (1500 × g, 10 min), and the absorbance of the supernatant was measured at 540 nm using a microplate reader. The hemolysis rate was calculated using the following formula:

​$\text{Hemolysis ratio }\left( \text{\%} \right)\text{=}\frac{\text{A}_{\text{sample}\text{​}}\text{-}\text{A}_{\text{negative control}}}{\text{A}_{\text{positive control}}\text{-}\text{A}_{\text{negative control}}}\text{×100}$​

***Cell Proliferation, Migration and Tube Formation Assays:*** For wound scratch assay, HUVECs and HaCat cells (1 × 10^5^/well) were seeded in a 6-well plate. The wound was scratched using a 1 mL pipette tip and washed with PBS to remove detached cells. Then the cells were cultured with PLGA (20 μg/mL) and artM (20 μg/mL), respectively. After incubation for 12 h, HUVECs and HaCat cells were stained with Calcein-AM/PI (0.1 %) for 30 min and imaged by fluorescence microscope (Leica DMBR, USA). For transwell assay, HUVECs (1 × 10^4^/well) were seeded in the upper chamber of a 24-well transwell plate. The lower chamber was added with 400 μL culture medium with 100 μL PLGA and artM, respectively. After incubation for 24 h, the migrated cells on the lower chamber were stained with crystal violet solution (0.5 %, Solarbio, G1062) for 10 min and photographed. For tube formation assay, 200 μL Matrigel (BD, 356234) was added into a pre-cooled 24-well plate and incubated for 30 min at 37 °C. HUVECs (3 × 10^3^/well) were incubated with PLGA and artM, respectively. After incubation for 12 h, HUVECs were stained with Calcein-AM/PI solution and photographed by fluorescence microscope.

***In Vitro Macrophage Polarization Assay:*** BMDMs were isolated from C57BL/6 mice and seeded in 6-well plates as methods mentioned above. Then, BMDMs were treated with PBS, IL-4 (40 ng/mL), PLGA, PLGA-Lys (20 μg/mL) or artM (20 μg/mL) for 48 h. Then, BMDMs were collected and stained with FITC-conjugated anti-CD86 antibodies (BioLegend, Cat. No.105006), PE-conjugated anti-F4/80 antibodies (BioLegend, Cat. No. 111604) and APC-conjugated anti-CD206 (BioLegend, Cat. No.141708), and then analyzed by flow cytometry.

***Crosstalk of Macrophages and T Cells:*** Spleen lymphocytes were extracted from spleen of C57BL/6 mice using lymphocyte isolation fluid (Solarbio, Beijing, China). The collected lymphocytes were seeded on the inserted chamber with a pore size of 0.4 μm (Corning, Cat. No. 3450). PBS, BMDMs, M2 macrophages (IL-4-treated BMDMs), PLGA, and artM were added into the 6-well plates for co-culture with lymphocytes. After 48 h of co-culture, the lymphocytes were collected and stained with FITC-conjugated anti-CD3 (BioLegend, Cat. No. 100204), PE-conjugated anti-CD4 (BioLegend, Cat. No. 100408), and APC-conjugated anti-IL-4 (BioLegend, Cat. No.504106) antibodies according to the manufacturer’s guidelines, and then analyzed by flow cytometry.

***Western Blot:*** Cells membrane lysates were lysed by RIPA lysis buffer (Beyotime, Cat. No. P0013B) containing 1 mM PMSF. After incubation for 30 min on ice, and then centrifuged for 15 min (4 °C 12,000 rpm). Next, protein loading buffer (Beyotime, Cat. No. P0015) was added to the supernatant, and the protein sample was obtained by heating at 100 ℃ for 10min. Then, protein samples were separated by SurePAGE™ (GenScript, Cat. No. M00660) and transferred onto poly (vinylidene difluoride) (PVDF) membranes (0.45 μm). The membranes were blocked by bovine albumin (2 h) and then incubated with antibody over night at 4 ℃. After washing with TBST, the membranes were incubated with secondary antibodies for 2 h at room temperature. The proteins on the membranes were visualized by Chemiluminescence Imaging system (Tanon 5200, China). The primary antibody included anti-CD14 (1:1000, Abcam, Cat. No. ab221678), anti-CD120b (1:1000, ABclonal, Cat. No. A0387), anti-IL-10(1:1000, Abcam, Cat. No. ab310329), anti-CD11b (1:1000, ABclonal, Cat. No. A23508), anti-CD18(1:1000, ABclonal, Cat. No. A25810), anti-CD44 (1:1000, ABclonal, Cat. No. A24605) and anti-CD119(1:1000, ABclonal, Cat. No. A11653). The secondary antibody included HRP-labeled goat anti-mouse IgG (1:2000, Beyotime, Cat. No. A0216) and HRP-labeled goat anti-rabbit IgG (1:2000, Beyotime, Cat. No. A0208).

***Coomassie Brilliant Blue Staining:*** The protein sample preparation procedure is the same as above. At the end of the electrophoresis, the PAGE gel was incubated in Coomassie Brilliant Blue staining reagent (Solarbio, Cat. No. P1305). After dyeing, wash three times with the destaining solution for 4 h each time. Finally, the PAGE gel was stored in 5% acetic acid and photographed.

***Reverse Transcription Quantitative PCR (RT-qPCR):*** Total RNA was extracted from lymphocytes using the Total RNA Extraction Kit (Solarbio, Cat. No. R1200). cDNA was synthesized by using the HiScript III Reverse Transcriptase (Nanjing Vazyme Biotech Co., Ltd). AceQ Universal SYBR qPCR Master Mix (Nanjing Vazyme Biotech Co., Ltd) was used to perform quantitative real-time PCR. Relative gene expression was calculated by the comparative Ct (threshold cycle) method and normalized to the gene expression of β-actin. Primer sequences (forward and reverse) are listed in Table S1.

***In Vivo Degradation*** ***Analysis:*** Cy5-labeled artM (2 mg/mL, 100 μL) was subcutaneously injected into the back of BALB/c nude mice (female, 5-weeks-old). Fluorescence imaging was performed using a Maestro In-Vivo Imaging System (PerkinElmer, Waltham, MA, USA) with excitation at 640 nm and emission at 670 nm.

***Immunofluorescence and Immunohistochemistry for Tissue:*** Skin tissues were harvested and fixed with 4% paraformaldehyde, embedded in paraffin, and sliced into 5 µm thick sections. For immunofluorescence, the sections were incubated with the primary antibodies of Mouse anti-CD68 (1:200, Abcam, ab955), a rabbit polyclonal antibody to CD206 (1:200, Cell Signaling Technology [CST], 91992S), a rabbit polyclonal antibody to CD31(1:100, Abcam, ab222783), and a mouse polyclonal antibody to *α*-SMA (1:200, Abcam, ab240654). After incubation overnight, sections were incubated with Alexa Fluor 488 goat anti-rabbit IgG (1:500, CST, #4412) and Alexa Fluor 594 goat anti-mouse IgG (1:200, Affinity, #S0012) corresponding to the primary antibody. Images were observed by CLSM and quantitatively analyzed by ImageJ software.

For immunohistochemistry, the sections were incubated with the primary antibodies of a rabbit antibody to anti-VEGFA (1:100, Abcam, ab52917). After incubation overnight, sections were incubated with Goat Anti-Rabbit IgG (1:2000, Abcam, ab205718). Sections were incubated at room temperature for a duration of 50 min. Subsequently, wash the sections using PBS, repeating this process three times with each wash lasting for 5 min. Once the sections were slightly dry, they were stained by DAB Substrate kit for 15 min (Solarbio, DA1010). After that, rinsed the sections thoroughly with tap water. Next, re-stained the sections with hematoxylin for approximately 5 min. Finally, the sections were soaked in 1% ammonia for 1 min. Then, rinsed the sections thoroughly with tap water. Images were observed by microscope and quantitatively analyzed by ImageJ software.

***In Vivo Macrophage Polarization Analysis:*** Mouse skin tissues were dissected, minced with surgical scissors, and digested in 1.8 mL of digestion buffer (RPMI-1640 medium supplemented with 800 U/mL collagenase IV, 1 mg/mL dispase II, and 10 μg/mL DNase I). The mixture was incubated in a shaking incubator at 37 °C and 90 rpm for 90 min. Digestion was quenched by adding 1.8 mL of RPMI-1640 medium containing 10% fetal bovine serum (FBS). The resulting suspension was filtered through a 70 μm cell strainer to obtain a single-cell suspension. The single-cell suspension was stained with the following fluorescently labeled antibodies: FITC-conjugated anti-CD86 (BioLegend, 105006), PE-conjugated anti-F4/80 (BioLegend, 123110), and APC-conjugated anti-CD206 (BioLegend, 141708). After staining, the cells were analyzed by flow cytometry to determine the expression of macrophage surface markers.

***Cytokine Array Analysis:*** To assess the expression of pro-inflammatory cytokines at the wound site, fresh mouse skin tissue was dissected and minced with surgical scissors. The minced tissue was then homogenized in RIPA buffer containing protease inhibitors using a homogenizer (Servicebio, WE-C6). The homogenate was centrifuged at 12,000 × g for 15 min at 4 °C, and the supernatant was collected. Finally, the levels of inflammation-related cytokines in the tissue lysate were detected using a cytokine array kit (Abcam, ab133999) according to the manufacturer’s instructions.

***Genomic Analysis:*** The total RNA within the harvested skin tissue was extracted and subsequently measured using the NanoDrop 2000 Spectrophotometer (manufactured by Thermo Fisher Scientific). For the construction of a sequencing library, one to two μg total RNA from 3 each sample was utilized. The quality of this sequencing library was then detected by the Agilent 2100 Bioanalyzer (from Agilent Technologies). Subsequently, differentially expressed genes (DEGs) were screened based on the established criteria (p-value < 0.05 and |logFC| > 1). R language software (v4.0.0) was employed to construct a volcano plot and conduct functional enrichment analyses. These analyses encompassed the KEGG pathway analysis, GO term enrichment, and Gene Set Enrichment Analysis (GSEA). In addition, a PPI network was constructed for the DEGs to analyze the interactions between the proteins encoded by these genes. Functional annotation of all identified proteins was performed using GO (http://geneontology.org/) and KEGG pathway (http://www.genome.jp/kegg/). Protein-protein interaction analysis was performed using the String v11.5.


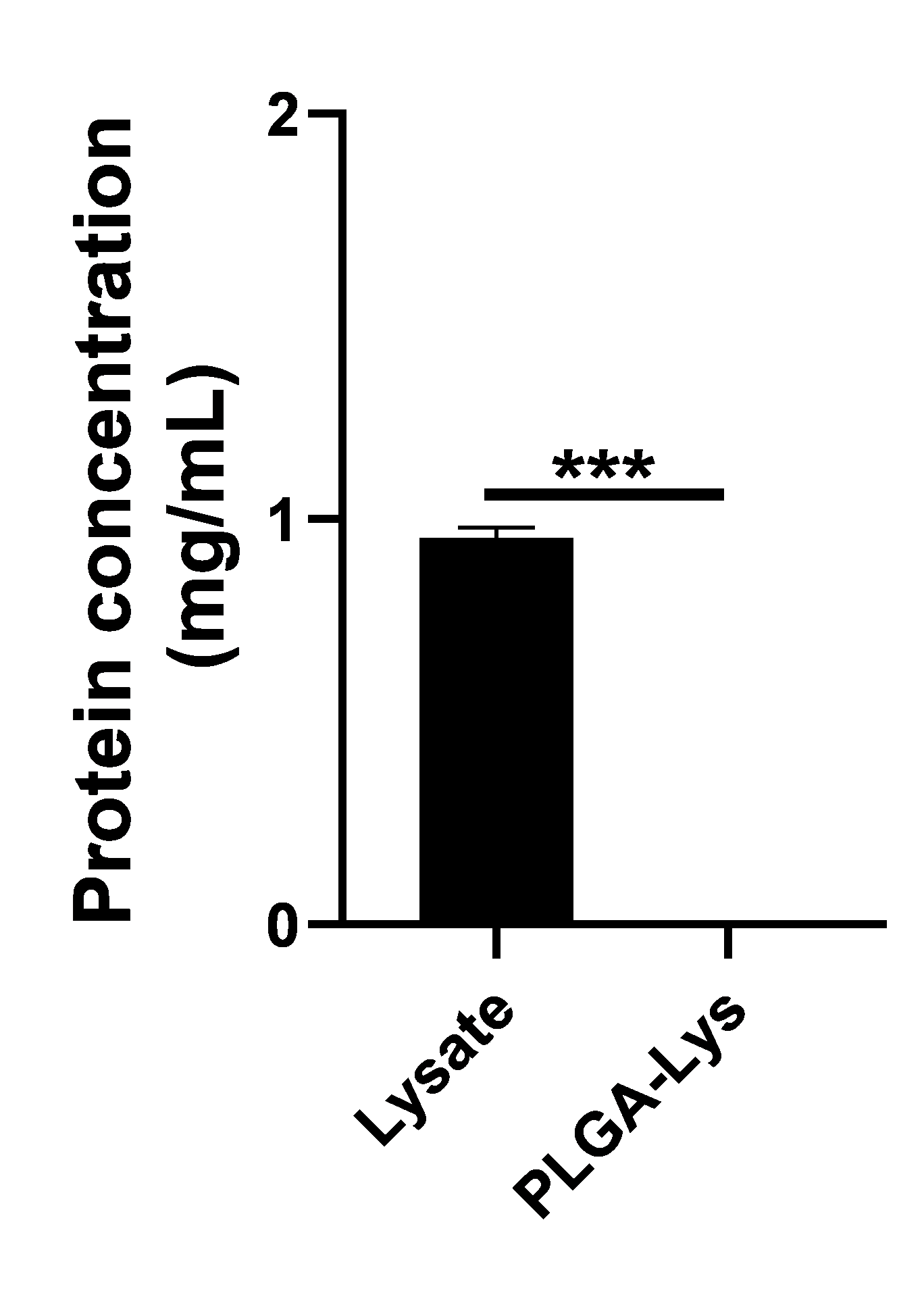


**Figure S1.** Free protein concentrations in lysate and PLGA-Lys supernatants post-centrifugation.

**
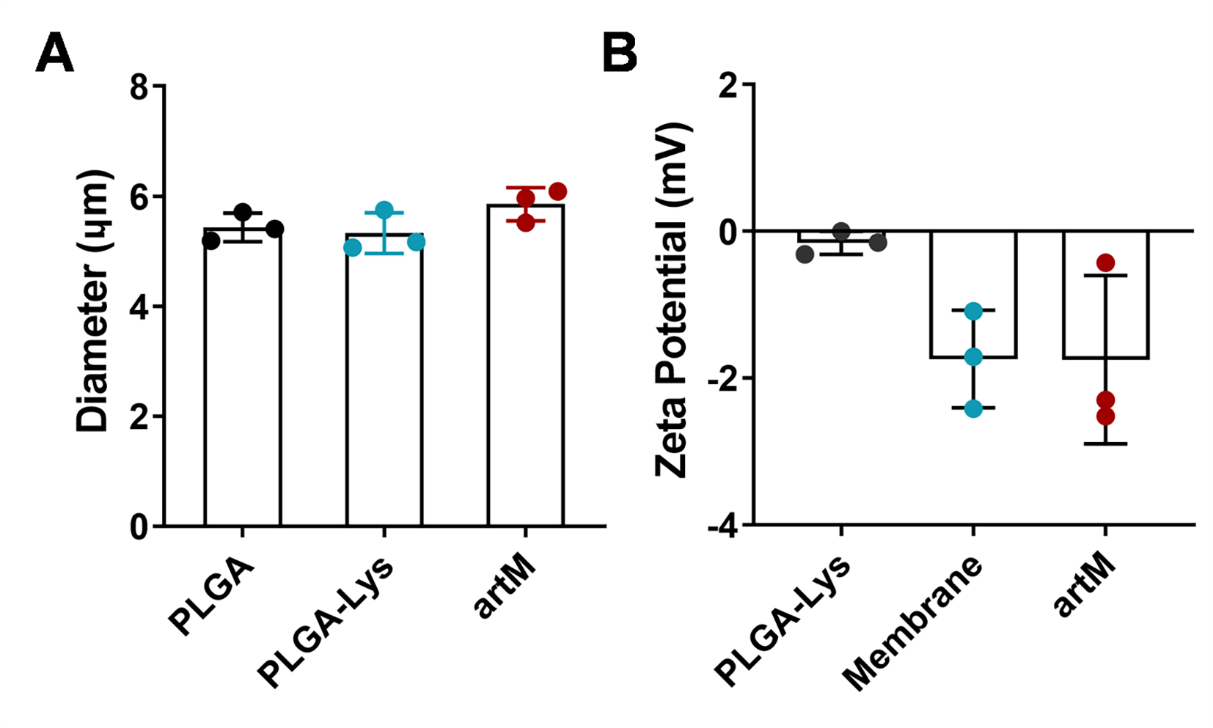
**

**Figure S2.** A) Diameter of the artM determined by DLS at the concentration of 0.1 mg/mL. B) Zeta potential of the PLGA-Lys, macrophage cell membrane and the artM.

**
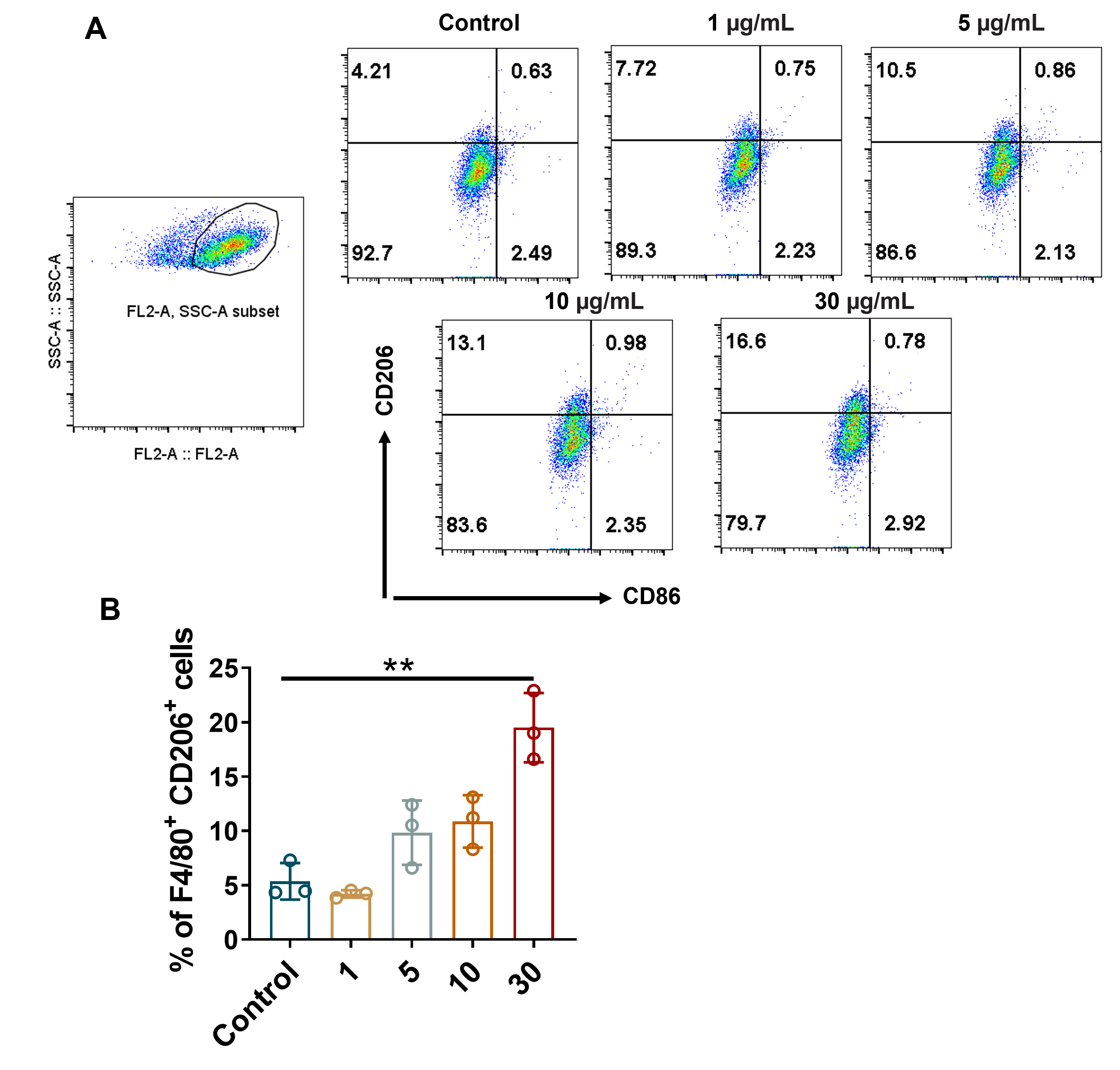
**

**Figure S3.** A) Flow cytometry analysis of CD206 (M2 marker) and CD86 (M1 marker) expression of BMDMs after receiving different concentration of lysate proteins treatment (gated on F4/80^+^ cells). B) Percentage of M2 (F4/80^+^CD206^+^) type macrophages (n = 3).


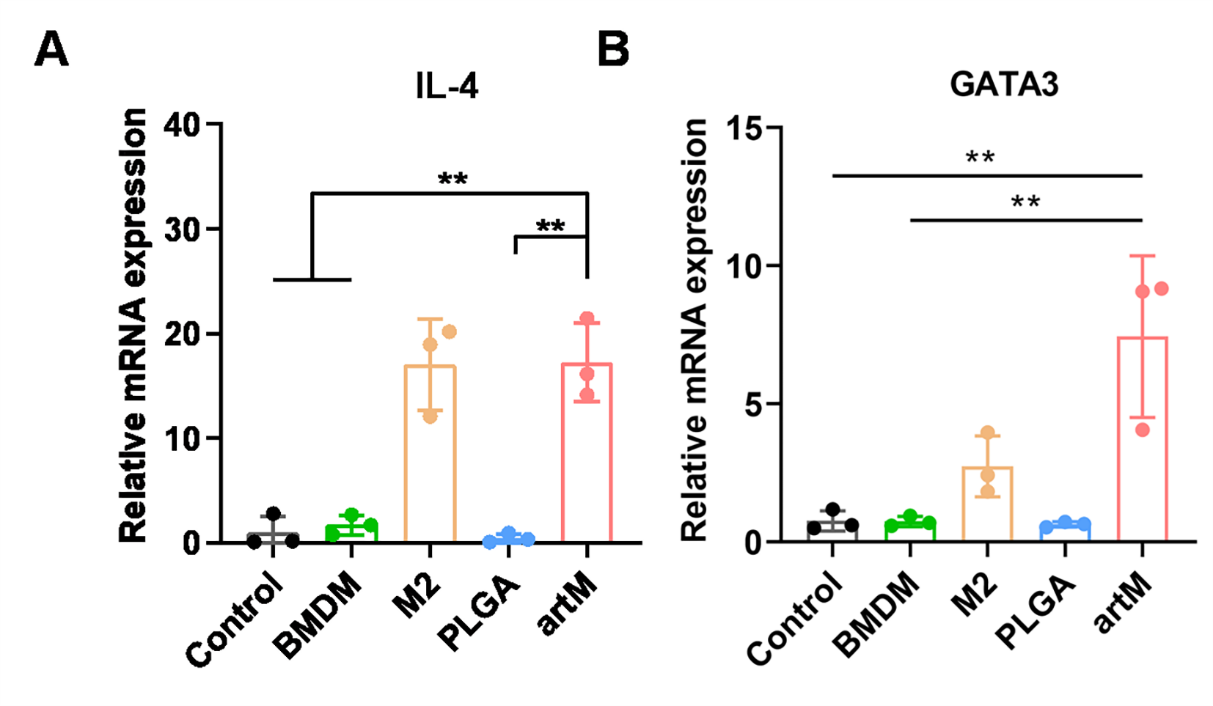


**Figure S4.** The mRNA expression of *IL-4* (A) and *GATA3* (B) in activated lymphocytes (n = 3).

**
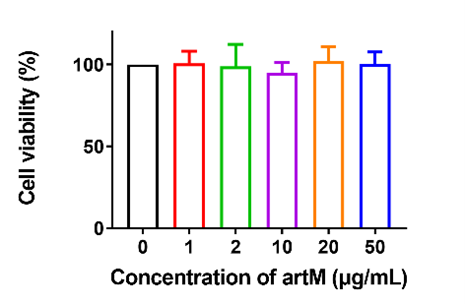
**

**Figure S5.** The viability of L929 cells treated with artM for 24 h was evaluated by CCK-8 assay (n = 5).


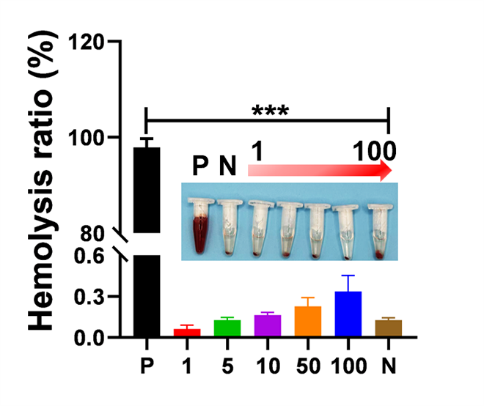


**Figure S6.** Hemolytic activity of artM was quantified at various concentrations (1-100 μg/mL n = 3). P: Positive control (water); N: Negative control (saline).

**
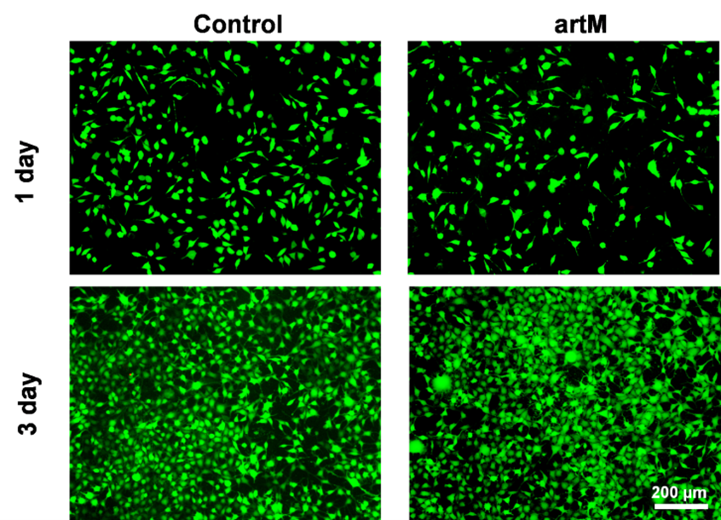
**

**Figure S7.** Live/dead staining of L929 cells treated with artM for 3 days. Scale bar: 200 μm.


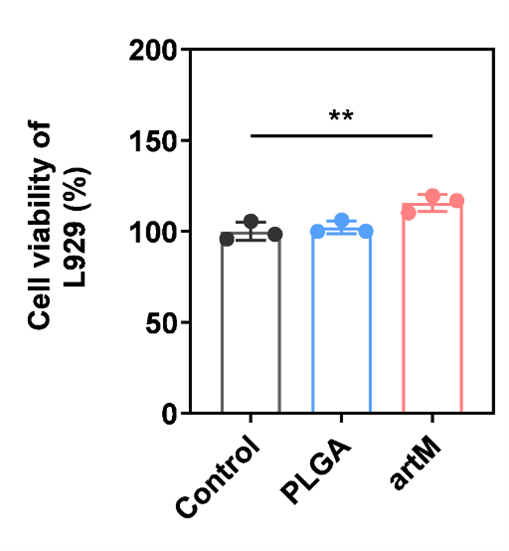


**Figure S8.** The cell viability of L929 cells treated with different formulations for 24 h determined by CCK-8 kit (n = 3).


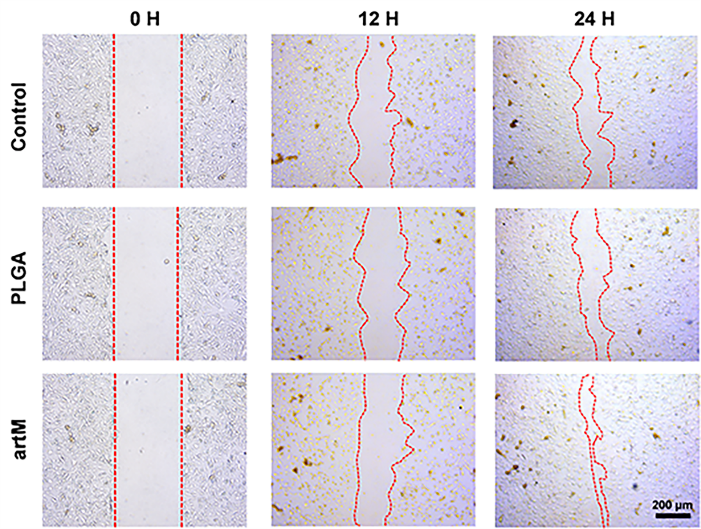


**Figure S9.** HUVEC migration after treatment with different formulations for 24 h. Scale bar: 200 μm.


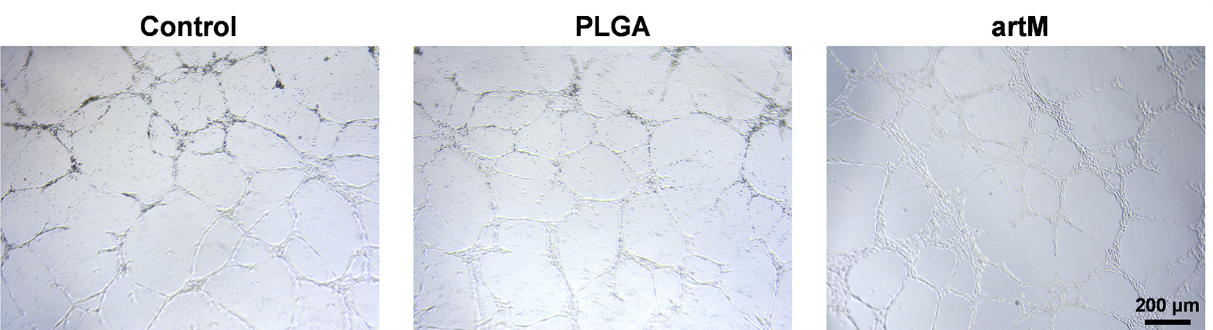


**Figure S10.** Representative images of the tube formation of HUVECs treated with different formulations for 12 h. Scale bar: 200 μm.

**
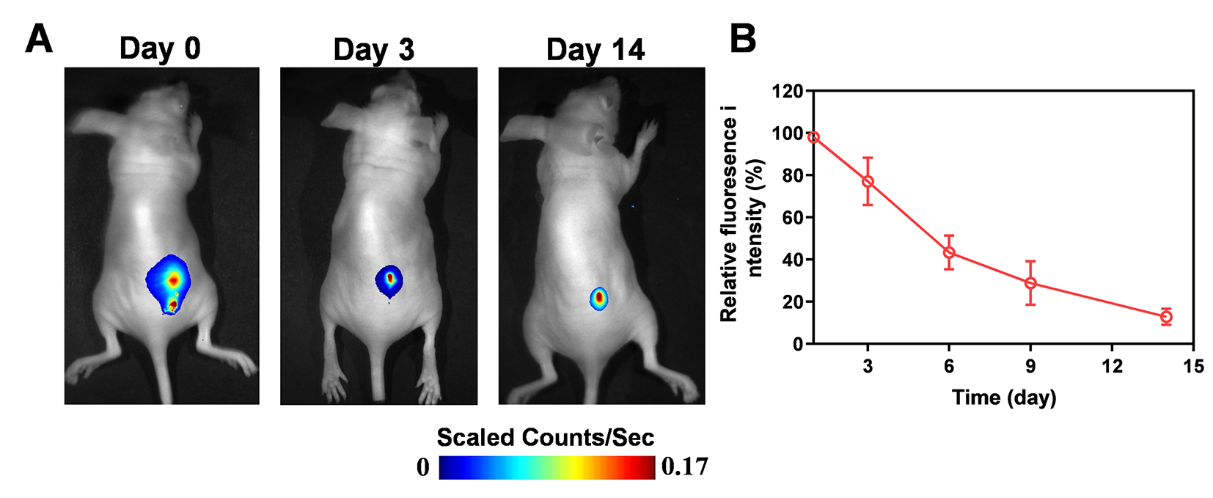
**

**Figure S11.** A) Time-dependent in vivo fluorescent images of mice after subcutaneous injection with Cy5-labeled artM. B) The fluorescence intensity of Cy5 at different time points after injection.


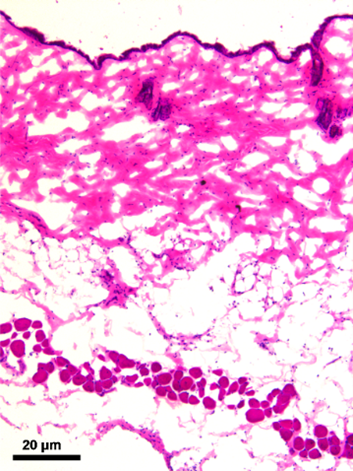


**Figure S12.** H&E staining images of the DTPI model at Day 0 post-modeling Scale bar: 20 μm.


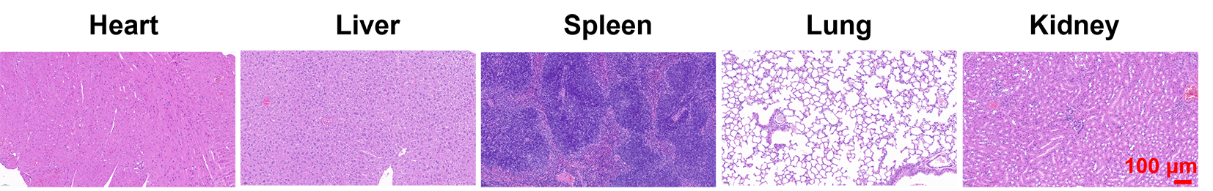


**Figure S13.** Representative images of H&E staining of the heart, liver, spleen, lung and kidney on day 14. Scale bar: 100 μm.


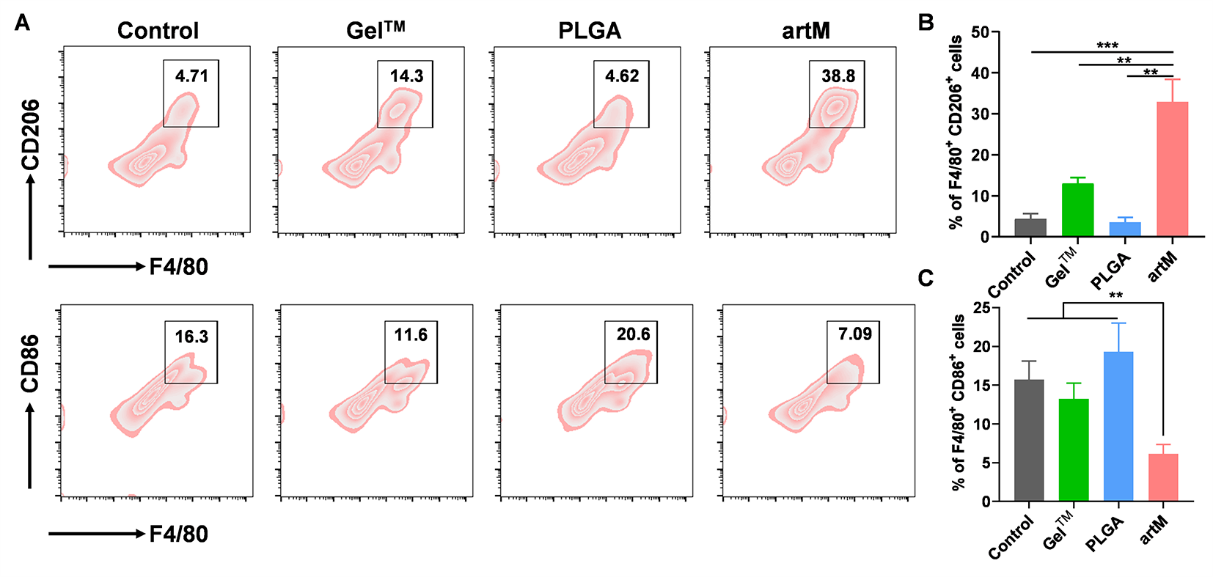


**Figure S14.** A) Flow cytometric analysis of M1 (CD86^+^) and M2 (CD206^+^) macrophages in wound tissues on day 14. B-C) Quantitative analysis of the proportions of B) M2 and C) M1 macrophages within total macrophages (F4/80^+^) (n = 3).


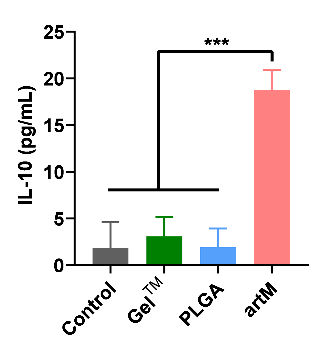


**Figure S15.** IL-10 analyzed by ELISA (n = 4).


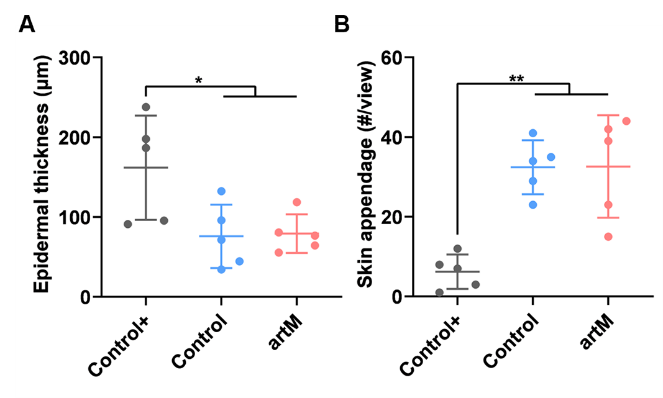


**Figure S16.** A-B) Quantitative analysis of epidermal thickness A) and skin appendages regeneration B) at the wound site for each group at day 14 (n = 5).

**
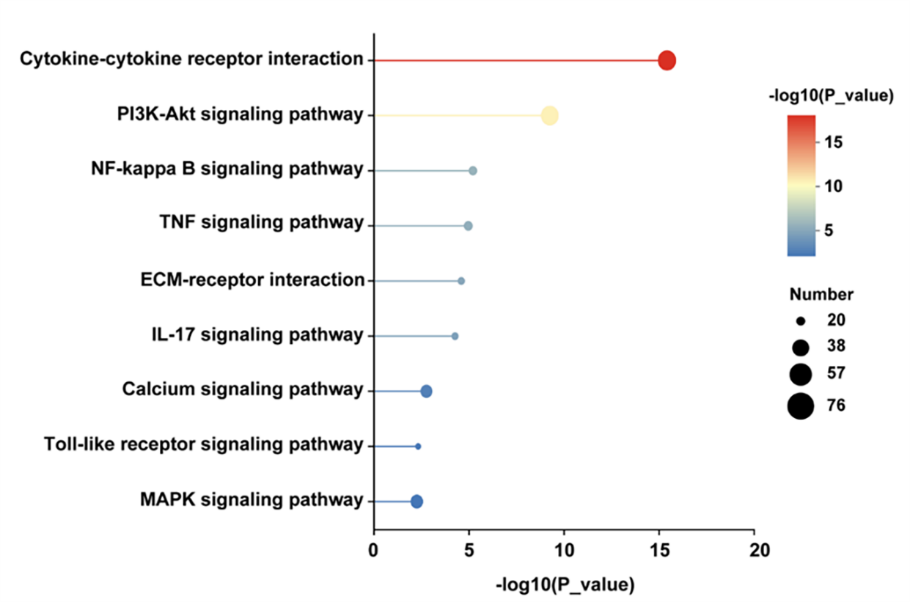
**

**Figure S17.** KEGG pathway enrichment analysis of DEGs. KEGG, Kyoto Encyclopedia of Genes and Genomes; DEGs, differentially expressed genes.

**
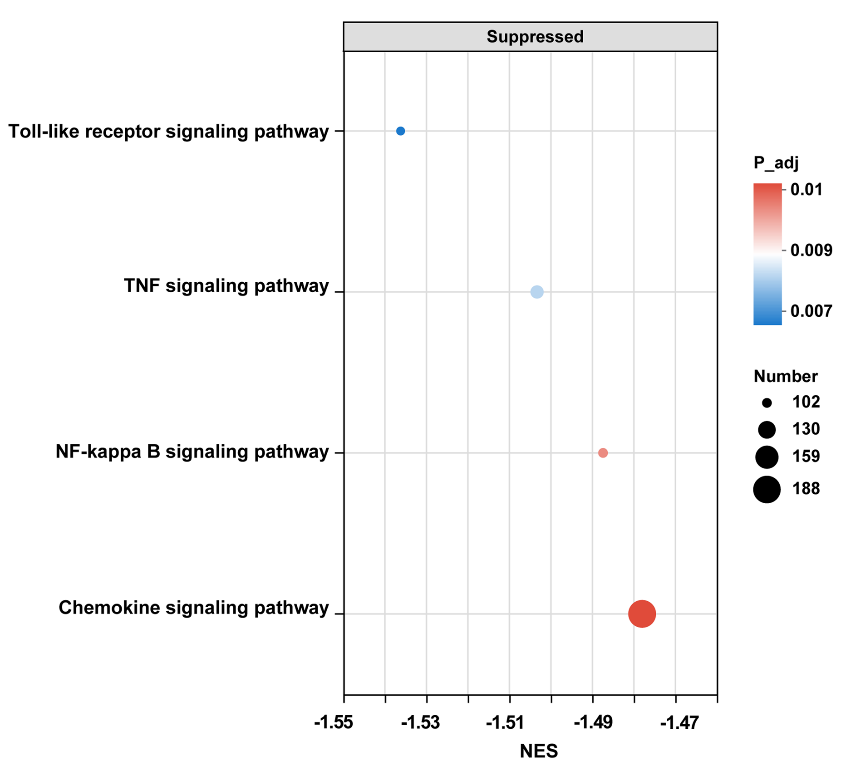
**

**Figure S18.** Gene Set Enrichment Analysis (GSEA) of DEGs.

**
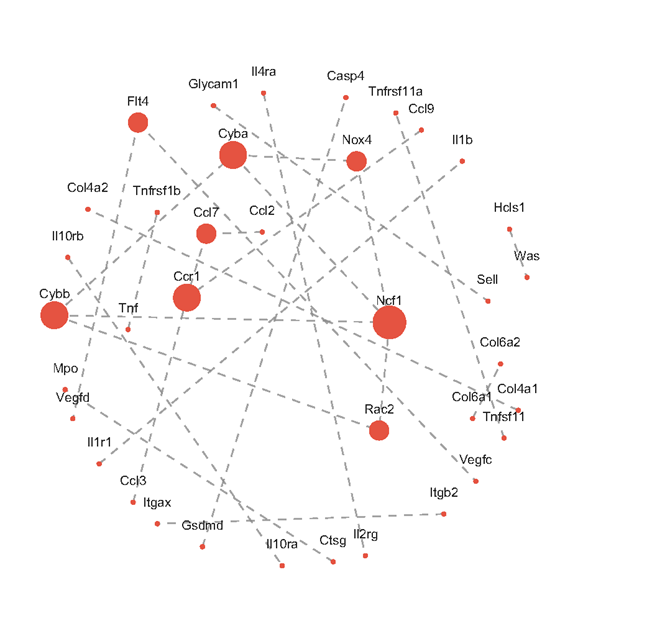
**

**Figure S19.** Protein-Protein Interaction (PPI) network of top 25 highly associated DEGs.

**Table S1.** Primer sequences

| Primer | Forward (5' - 3') | Reverse (5' - 3') |
| --- | --- | --- |
| IL-4 | ATGGATGTGCCAAACGTCCT | AAGCCCGAAAGAGTCTCTGC |
| STAT6 | GAGCTACTGGTCAGATCGGC | GGATGACGTGTGCAATGGTG |
| JAK1 | CGTCAAACCTGTGTCTCGCT | CCCCCAAAGTCTACGCTGTT |
| GAT3 | CCCTTATCAAGCCCAAGCGA | GTCAGGGGTATTATGAAGCTTGT |
| CD206 | gTgg9gACCTggCAAgTATC | CACTggggTTCCATCACTCC |
| Arg-1 | ACATTGGCTTGCGAGACGTA | ATCACCTTGCCAATCCCCAG |
| TGF-β | ACTGGAGTTGTACGGCAGTG | GGGGCTGATCCCGTTGATTT |
| IL-10 | CCAAggTgTCTACAAggCCA | gCTCTgTCTAggTCCTggAgT |
| CD86 | CTTACggAAgCACCCACgAT | CggCAgATATgCAgTCCCAT |
| VEGFa | TGGGAGAACCCAAATGCTCC | CACTAGGCAACAGCACCTCA |
| β-actin | TCTGTGTGGATTGGTGGCTCTA | CTGCTTGCTGATCCACATCTG |
